# Korea hypertension fact sheet 2022: analysis of nationwide population-based data with a special focus on hypertension in the elderly

**DOI:** 10.1186/s40885-023-00243-8

**Published:** 2023-08-15

**Authors:** Hyeon Chang Kim, Hokyou Lee, Hyeok-Hee Lee, Gabin Lee, Eunji Kim, Moses Song, Jenny Moon, Yeeun Seo, for the Korean Society of Hypertension (KSH)-Hypertension Epidemiology Research Working Group

**Affiliations:** 1 Department of Preventive Medicine, Yonsei University College of Medicine, Seoul, Republic of Korea; 2 Institute for Innovation in Digital Healthcare, Yonsei University Health System, Seoul, Republic of Korea; 3 Department of Public Health, Yonsei University Graduate School, Seoul, Republic of Korea

**Keywords:** Hypertension, Prevalence, Awareness, Therapeutics, Elderly, Korea

## Abstract

**Background:**

The Korean Society of Hypertension has published the Korea Hypertension Fact Sheet 2022 to provide an overview of the magnitude and management status of hypertension and their recent trends.

**Methods:**

The Fact Sheets were based on the analyses of Korean adults aged 20 years or older of the 1998–2020 Korea National Health and Nutrition Examination Survey and the 2002–2020 National Health Insurance Big Data.

**Results:**

As of 2020, 29.4% of the adult population aged 20 or older in Korea, about 12.6 million people, have high blood pressure, of which 5.0 million (40%) are 65 years of age or older and 1.2 million (10%) are 80 years of age or older. Among those with hypertension, the awareness rate is 69%, the treatment rate is 65%, and the control rate is 47%. The number of people diagnosed with hypertension increased from 3.0 million in 2002 to 10.5 million in 2020. During the same period, the number of people using antihypertensive medication increased from 2.5 million to 9.9 million, and the number of people adherent to treatment increased from 0.6 million to 7.4 million. Among those treated for hypertension in 2020, 74% used angiotensin blockers, 61% used calcium channel blockers, 24% used diuretics, and 15% used beta blockers. Combination therapy with at least two classes of antihypertensive medication consisted of 60% of all antihypertensive prescriptions. The number of people with hypertension aged 65 or older is increasing very rapidly compared to those aged 20–64. Awareness and treatment rates of hypertension improved rapidly, especially in those aged 65 or older, but the rate of improvement slowed since 2012.

**Conclusions:**

In Korea, the level of hypertension management is improving, but the absolute number of people with hypertension, especially elderly hypertension, is increasing due to the rapid aging of the population. It is necessary to develop more efficient and target-specific policies to control blood pressure and prevent cardiovascular disease.

**Supplementary Information:**

The online version contains supplementary material available at 10.1186/s40885-023-00243-8.

## Background

Hypertension, or elevated blood pressure, is a medical condition that significantly increases the risks of heart, brain, kidney and other diseases. Hypertension is a major cause of premature death worldwide [1, 2]. It is estimated that the number of people with hypertension doubled from 648 million in 1990 to 1,278 million in 2019, and only 38% of male hypertension patients and 47% of female hypertension patients are diagnosed and treated [3]. Although the age-adjusted cardiovascular disease mortality rate has been decreasing in Korea; heart disease and stroke still remain among the most common causes of death [4]. Moreover, due to the rapid aging of the population, the absolute number of people with hypertension and cardiovascular disease is expected to increase [5]. According to the National Health Insurance Service (NHIS) statistics in Korea, the estimated medical cost of treating hypertension was 4.3 trillion Korean Won, accounting for 4.5% of all medical expenses or 10.9% of medical expenses for chronic diseases [6]. Hitherto, controlling blood pressure is crucial not only to reduce the burden of disease at a societal level but also to improve the quality of life at an individual level. Continuous monitoring of hypertension prevalence and management status should be the first step in reducing its burden. To achieve this, the Korean Society of Hypertension had published its first Hypertension Fact Sheet in 2018, and have been periodically updating it thereafter [7–9].

## Methods

The Korea Hypertension Fact Sheet 2022 analyzed two nationally representative datasets. The first one is the Korea National Health and Nutrition Examination Survey (KNHANES) from 1998 to 2020. The KNHANES is a national surveillance system in Korea that assesses the health and nutritional status of noninstitutionalized Korean population since 1998 [10, 11]. The second one is the National Health Insurance (NHI) Big Data from 2002 to 2020. Organized by the NHIS, the NHI Big Data contains socio-demographics, hospital claims with International Classification of Diseases, 10th Revision (ICD-10, I10) coding, and mortality data of the entire population of Republic of Korea [12]. Previously, the Korea Hypertension Fact Sheet 2018 analyzed adults aged 30 years from the KNHANES data and people of all age in the NHI Big Data. Since the Korea Hypertension Fact Sheet 2020, both NHANES and NH-BD were analyzed for adult data aged 20 or older [8]. The English version of the “Korea Hypertension Fact Sheet 2022” is attached as a supplementary material of this manuscript. The Korean version is available at http://www.koreanhypertension.org/reference/guide.

### Analysis of the KNHANES from 1998 to 2020

There have been 8 rounds of KNHANES between 1998 and 2018: KNHANES I (1998), KNHANES II (2001), KNHANES III (2005), KNHANES-IV (2007–2009), KNHANES V (2010–2012), KNHANES VI (2013–2015), KNHANES VII (2016–2018), and KNHANES VIII (2019–2021). However, only a part of the 8th round data (year 2019 and 2020) were available for this report. Hypertension was defined as systolic blood pressure (SBP) ≥ 140 mmHg, diastolic blood pressure (DBP) ≥ 90 mmHg, or self-reported use of antihypertensive medication for the purpose of blood pressure control. Awareness rate was defined as the proportion of people with physician diagnosis of hypertension among all people with hypertension. Treatment rate was defined as the proportion of people using antihypertensive drugs for 20 days or more per month among all people with hypertension. Control rate was defined as the proportion of people with SBP < 140 mmHg and DBP < 90 mmHg among all people with hypertension and people treated for hypertension [13]. To evaluate the magnitude and management status of hypertension without the effects of population aging, age-standardized rates were calculated based on the demographics of the Korean population in 2005 according to the Population and Housing Census, Statistics Korea. To take into account the effect on estimator variance attributable to the KNHANES’ stratified multistage clustered probability sampling design, we applied survey sampling weights to all the analyses.

### Analysis of the NHI Big Data from 2002 to 2020

While the KNHANES data analysis defined hypertension based on measured blood pressure levels and use of antihypertensive medication, the NHI Big Data analysis defined hypertension based on diagnosis codes, because the claim database did not have records of blood pressure measurements. Healthcare utilization was defined as at least one health insurance claim for diagnosis of essential hypertension (I10) each year. Treatment of hypertension was defined as at least one health insurance claim for hypertension diagnosis with antihypertensive drug prescription each year. Adherence to treatment was defined as receiving prescriptions of antihypertensive drugs ≥ 290 days (80%) each year. Antihypertensive drugs were classified into diuretics (DU, thiazide-related and loop diuretics), beta-blockers (BB), calcium channel blockers (CCB), angiotensin-converting enzyme inhibitors (ACEi), angiotensin receptor blockers (ARB), potassium-sparing diuretics (PSD), or others (alpha-blockers, vasodilators, etc.). If the regimen of antihypertensive drug had switched in a year, one with the longest duration was selected as the representative prescription of the patient for the given year. For the analyses of hypertension in the elderly, the population aged 65 or older was classified as the elderly, and this group was further classified into age 65–79 years and age 80 years or older.

## Results

### Trends of average blood pressure and hypertension prevalence

The average blood pressure of Korean adults has decreased between 1998 and 2008, but there has been little change in the last 12 years. Population mean SBP/DBP level was 118/76 mmHg for Korean adults aged 20 years or older (Supplement, page 8). Over the last 20 years, the age-standardized mean blood pressure levels have decreased yet without significant change in recent years. The age-standardized prevalence of hypertension among adults aged 20 years or older also modestly decreased from 26.0% (men 29.6%, women 22.3%) in 1998 to 23.3% (men 29.1%, women 17.0%) in 2020 (Supplement, page 10). Over the same period, the age-standardized prevalence of hypertension among adults aged 30 years or older decreased from 30.7% (men 33.4%, women 27.4%) to 28.4% (men 34.9%, women 21.3%) (Supplement, page 11). However, with the rapid aging of the population, the absolute number of people with hypertension has steadily increased; 12.6 million Korean adults have hypertension as of 2020. In particular, the number of elderly women with hypertension has increased rapidly. In 2020, estimated people with hypertension was 5.15 million men and 2.49 million women under the age of 65, but 2.00 million men and 3.01 million women aged 65 years or older. Moreover, among those with hypertension aged 80 or older, 301 thousands were male and 922 thousands were female (Supplement, page 13).

### Trends of hypertension management

In general, the hypertension management (awareness, treatment, and control rates) has improved significantly over the past two decades. In 2020, among adults aged 20 or older with hypertension, the awareness rate was 69.5%, the treatment rate was 64.8%, and the control rate was 47.4%. However, the degree of management varied greatly by age and sex. All management indices tended to be higher in older adults than in young adults, and higher in women than in men. However, gender-difference varies depending on age. Women under the age of 50 have higher awareness, treatment, and control rates compared to men of same age. After the age of 60, the awareness and treatment rates become similar in men and women, and the control rate is even lower in women than in men (Supplement, pages 16–19).

### Healthcare utilization for hypertension

The number of people diagnosed with hypertension has increased 3.5 times from 3.0 million in 2002 to 10.5 million in 2020. People receiving antihypertensive medications also increased 3.9 times from 2.5 million in 2002 to 9.9 million in 2020. More importantly, the number of people adherent to antihypertensive medication has increased more than twelvefold from 0.6 million in 2002 to 7.4 million in 2020 (Supplement, page 22). Of the 9.9 million people receiving treatment for hypertension, 58.4% were receiving dyslipidemia treatment, 27.1% also receiving diabetes treatment, and 21.2% were receiving treatment for both dyslipidemia and diabetes (Supplement, page 23). The use of combination therapy has rapidly increased, with 40.1% using one class, 43.6% using two classes, and 16.3% using three or more classes of antihypertensive drug in 20,202 (Supplement, page 24). In 2020, the most prescribed antihypertensive drug class was ARB (73.8%), followed by CCB (61.4%), DU (24.2%), BB (15.5%), PSD (1.8%) and ACEi (1.6%) (Supplement, page 25). The most frequently prescribed regimen for hypertension treatment was dual therapy of ACEi/ARB plus CCB, followed by ARB monotherapy and CCB monotherapy (Supplement, page 26–27). Overall, the types of antihypertensive medication were not significantly different between men and women. However, when limited to age of 20–39, monotherapy was more common in women than in men (58.9% vs. 37.3%), and ACEi/ARB prescriptions were less common in women than in men (55.1% vs. 63.71.8%) (Supplement, page 28–29).

### Hypertension in the elderly

The proportion of the population aged 65 or older in total adult hypertension was 22.5% in 1998, but it surpassed 40% in 2019, and 39.6% in 2020. Fortunately, in elderly hypertension, the proportion of treated and controlled hypertension increased rapidly, accounting for only 6.6% in 1998, but recently it has been approaching 60% (Supplement, page 31). The awareness rate, treatment rate, and treatment adherence rate have been improved more rapidly in the older people than in younger people with hypertension. However, it is also worth mentioning the recent decrease in awareness, treatment, and control rates among male hypertension patients aged 80 or older (Supplement, page 16–20).

## Discussion

The Korea Hypertension Fact Sheet 2022 provides an overview on the magnitude and management status of hypertension in Korea. Although the population average blood pressure and the prevalence of hypertension have remained relatively stable over the last decade, the absolute number of people with hypertension has increased steadily and now exceeds 12 million due to population aging. Of particular concern is the rapidly increasing number of elderly individuals with hypertension, with the number of very elderly women with hypertension increasing even faster. This trend may lead to a significant increase in the burden of hypertension and its complications. Fortunately, there have been improvements in the awareness, treatment, and treatment adherence rates among this age group. However, it is worth noting that the proportion of combination therapy use has recently decreased among older adults taking antihypertensive medications. One possible explanation for this trend is that increasing adherence to antihypertensive medication regimens may have led to sufficient blood pressure control with a single antihypertensive drug. Another possible explanation is that the target blood pressure has become more diverse among older patients. However, to fully understand the factors contributing to the decrease in combination therapy use among older patients, further studies with more detailed clinical information will be needed. The greatest novelty of the Korea Hypertension Fact Sheet lies on its generalizability; the KNHANES provides unbiased sampling of the Korean population, and the NHI Big Data provides medical service uses of the entire nation. However, there are some limitations to be addressed. First, the KNHANES is based on non-institutionalized residents of Korea; thus, it might not include people with severe diseases. Second, using the I10 code as an operational definition of hypertension was intended to maintain comparability across series-issued Hypertension Fact sheets. But we acknowledge that this approach has limitations for identifying people with hypertension, particularly for older patients with hypertensive complications or target organ damage. Third, the exact collection methods and survey details varied across the KNHANES. Despite standardized protocols and rigorous quality control procedures, such variability may have affected the analysis on secular trends. Fourth, the NHI Big Data may not be optimal for identifying disease occurrence and prevalence, because the data have been collected for medical service claims and reimbursement purposes. Fifth, the adherence to antihypertensive medication was evaluated on a prescription basis. Thus, it is possible that adherence was overestimated, because we cannot ascertain whether the drug was actually taken or not. Finally, we were limited to anonymized datasets; therefore, the data linkage between the two datasets was unachievable.

## Conclusions

Despite the significant improvement in hypertension management over the past few decades, there is still room for further improvement. Due to the rapid population aging and increasing lifespan, the absolute burden of hypertension and its complications will continue to increase. The number of elderly hypertensive patients and hypertensive patients with multi-comorbidity is expected to increase rapidly, so it is necessary to respond to these changes. To prevent complications and death from hypertension, emphasizing the importance of treatment adherence and blood pressure control remains a top priority. We also need to develop tailored prevention and management strategies that are appropriate for and inclusive of various demographics.

## Electronic supplementary material

Below is the link to the electronic supplementary material.

Supplementary Material 1

Supplementary Material 2

Supplementary Material 3

## Data Availability

The datasets used and/or analyzed during the current study are available from the corresponding author on reasonable request.

## Declarations

### Consortium

The Korean Society Hypertension–Hypertension Epidemiology Research Working Group. Hyeon Chang Kim, Song Vogue Ahn, Sun Ha Jee, Sungha Park, Hae-Young Lee, Min Ho Shin, Sang-Hyun Ihm, Seung Won Lee, Hokyou Lee, Jong Ku Park, Il Suh, and Tae-Yong Lee.

## Abbreviations

ACEi: angiotensin-converting enzyme inhibitor
ARB: angiotensin receptor blockers
BB: beta-blockers
CCB: calcium channel blockers
DBP: diastolic blood pressure
DU: diuretics
ICD-10: International Classification of Diseases, 10th Revision
KNHANES: Korea National Health and Nutrition Examination Survey
NHI: National Health Insurance
NHIS: National Health Insurance Service
PSD: potassium-sparing diuretic
SBP: systolic blood pressure
