# Supplementary material for: Korea hypertension fact sheet 2022: analysis of nationwide population-based data with a special focus on hypertension in the elderly
Source: Clin Hypertens. 2023 Aug 15;29:22. doi: 10.1186/s40885-023-00243-8 (PMC10426053; doi:10.1186/s40885-023-00243-8)
Supplement: Supplementary file 1 — Supplementary Material 1 [file 40885_2023_243_MOESM1_ESM.pdf]

KOREA HYPERTENSION FACT SHEET 2022

The Korean Society of Hypertension

대한고혈압학회
THE KOREAN SOCIETY OF HYPERTENSION

Methods

Korea National Health and Nutrition Examination Survey

Subjects		Adults aged 20 years or older / 1998 to 2020
Definition of Hypertension		① Systolic blood pressure (SBP) ≥140 mmHg, or<br>② Diastolic blood pressure (DBP) ≥90 mmHg, or<br>③ Taking antihypertensive medication
Hypertension Management	Awareness rate	Proportion of people with physician diagnosis of hypertension among those with hypertension
	Treatment rate	Proportion of people taking antihypertensive medication for ≥20 days/month among those with hypertension
	Control rate (among prevalent)	Proportion of people with SBP <140 mmHg and DBP <90 mmHg among those with hypertension
	Control rate (among treated)	Proportion of people with SBP <140 mmHg and DBP <90 mmHg among those taking antihypertensive medication

Korea National Health Insurance Big Data

Subjects		Adults aged 20 years or older / 2002 to 2020
Healthcare Utilization	Diagnosis	≥1 health insurance claim for hypertension diagnosis (ICD-10: I10) each year
	Treatment	≥1 health insurance claim for hypertension diagnosis and antihypertensive prescription each year
	Adherence	Antihypertensive prescription ≥290 days (80%) each year
Antihypertensive Treatment	Regimen	The combination of antihypertensive classes in a prescription; if the regimen is switched, one with the longest duration is selected for a given year
	Antihypertensive class	Diuretics (DU; thiazide and related diuretics, loop diuretics), Beta-blockers (BB), Calcium channel blockers (CCB), Angiotensin converting enzyme inhibitors (ACEi), Angiotensin receptor blockers (ARB), Potassium-sparing diuretics (PSD), Others (ETC; alpha-blockers, vasodilators, etc.)

Contents

Trends of Average Blood Pressure and Hypertension Prevalence

8 Trends of Average Blood Pressure
9 Trends of Average Blood Pressure by Age
10 Trends of Hypertension Prevalence
12 Trends of Hypertension Prevalence by Sex and Age
13 Trends of Number of People with Hypertension

Trends of Hypertension Management

16 Trends of Awareness Rate by Sex and Age
17 Trends of Treatment Rate by Sex and Age
18 Trends of Control Rate by Sex and Age

Healthcare Utilization for Hypertension

22 Trends of Healthcare Utilization for Hypertension
23 Trends of Co-Treatment for Dyslipidemia and Diabetes
24 Trends of Antihypertensive Medication Use
26 Composition of Antihypertensive Treatment
28 Antihypertensive Medication Use by Sex and Age

Hypertension in the Elderly

31 Trends of Number of People with Hypertension by Management Status
32 Trends of Awareness Rate in the Elderly
33 Trends of Treatment Rate in the Elderly
34 Trends of Adherence Rate in the Elderly
35 Trends of Combination Therapy Rate in the Elderly
36 Trends of Average Blood Pressure in the Elderly

Summary of Hypertension Statistics (Age 20+)

12.6 million
have hypertension

10.5 million
are diagnosed

9.9 million
are treated

7.4 million
are adherent
to treatment

Data Source: Korea National Health and Nutrition Examination Survey 2020, Korea National Health Insurance Big Data 2020

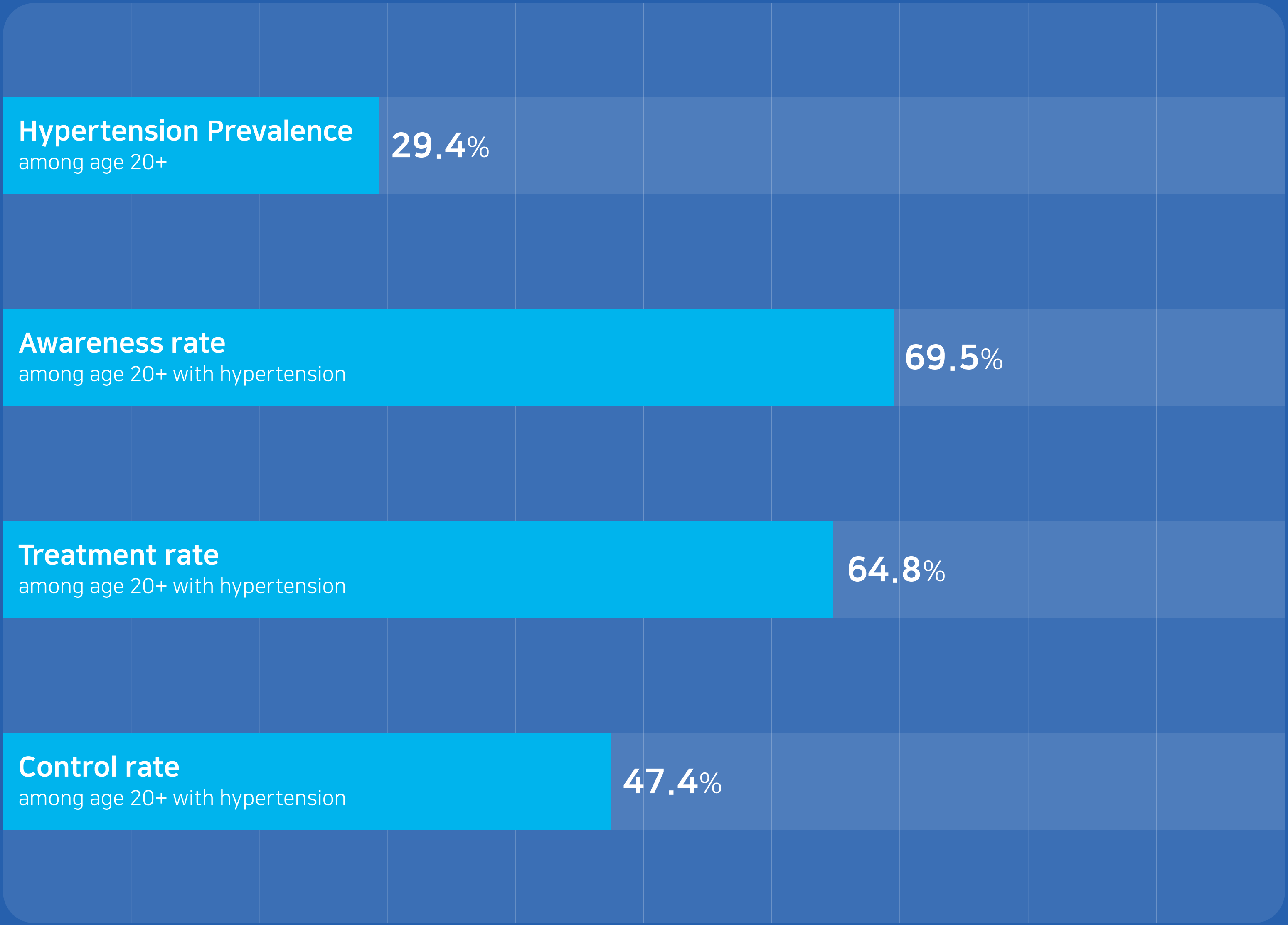

Data Source: Korea National Health and Nutrition Examination Survey 2020

KOREA HYPERTENSION
FACT SHEET 2022

Trends of Average Blood Pressure and Hypertension Prevalence

Trends of Average Blood Pressure

Trends of Average Blood Pressure by Age

Trends of Hypertension Prevalence

Trends of Hypertension Prevalence by Sex and Age

Trends of Number of People with Hypertension

Trends of Average Blood Pressure

(Age 20+, Age-standardized)

	Crude	Age-standardized
Age 20+	118/76 mmHg	116/76 mmHg
Age 30+	120/77 mmHg	118/77 mmHg

Systolic
(mmHg)

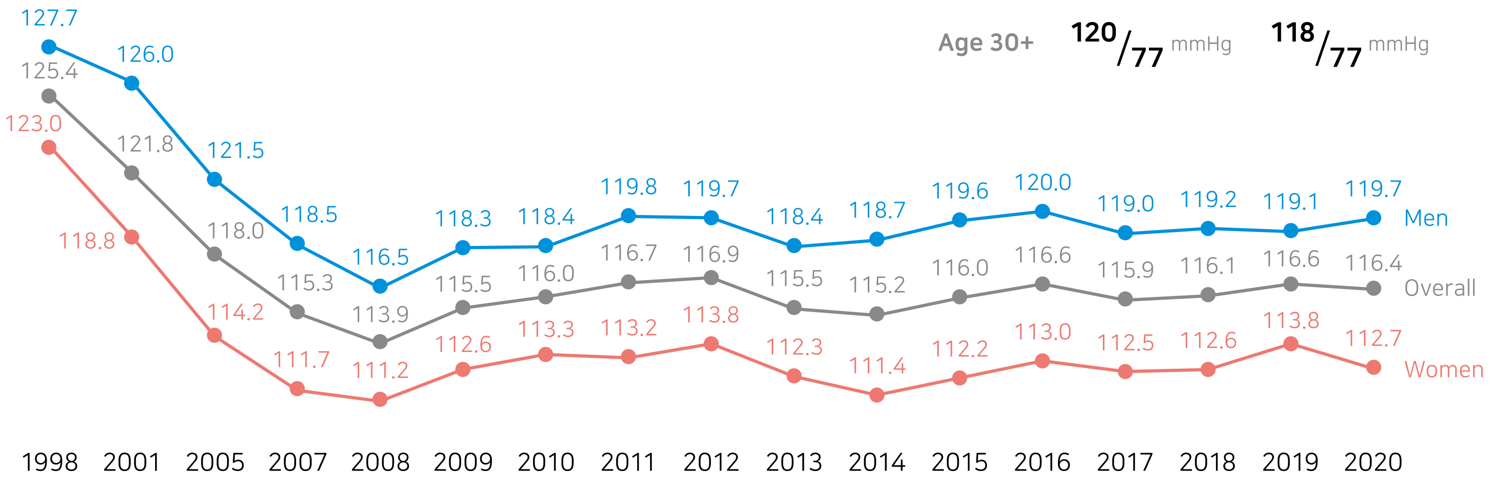

Diastolic
(mmHg)

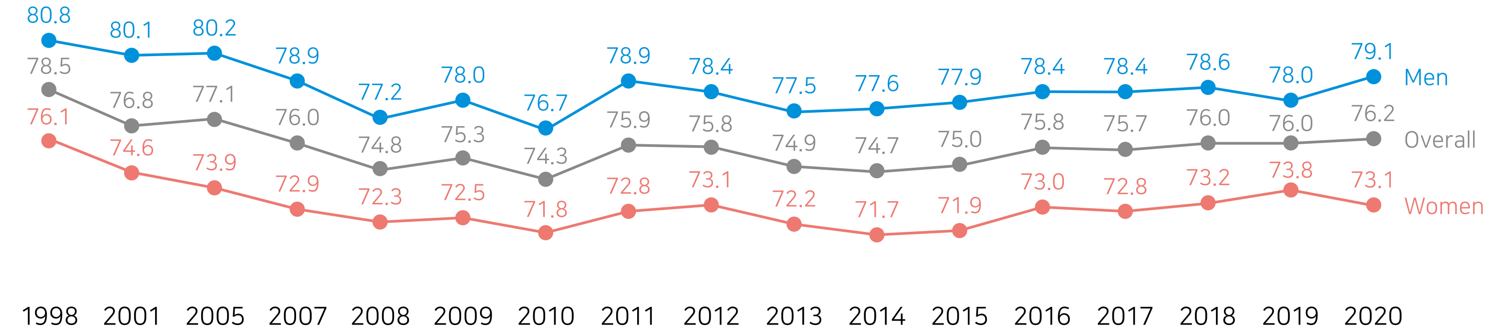

Data Source: Korea National Health and Nutrition Examination Survey 1998-2020
(Directly age-standardized to the 2005 projected population)

Trends of Average Blood Pressure by Age

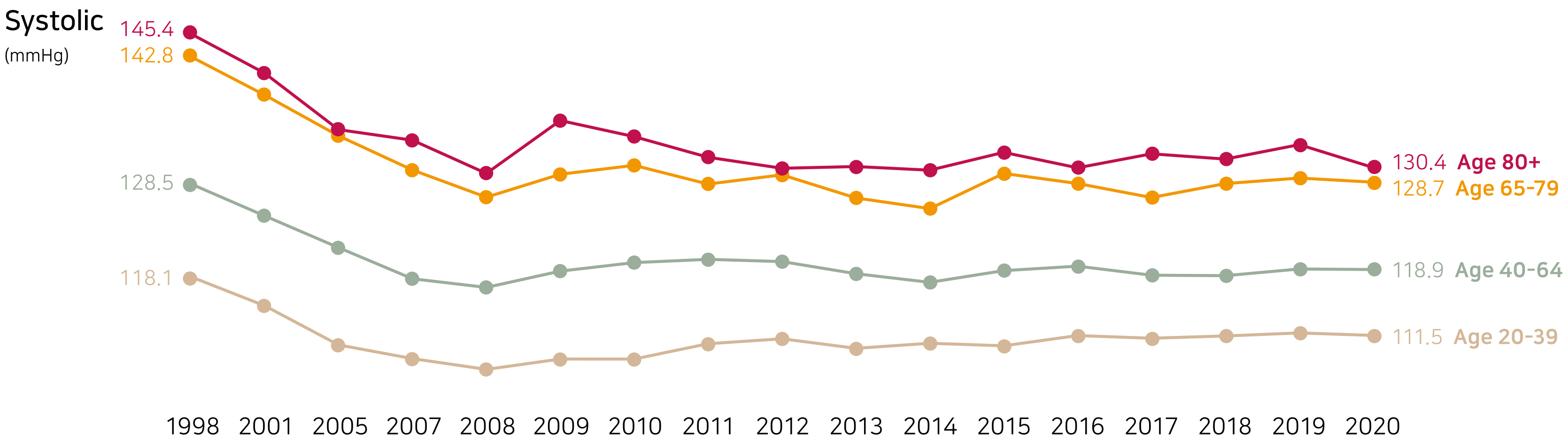

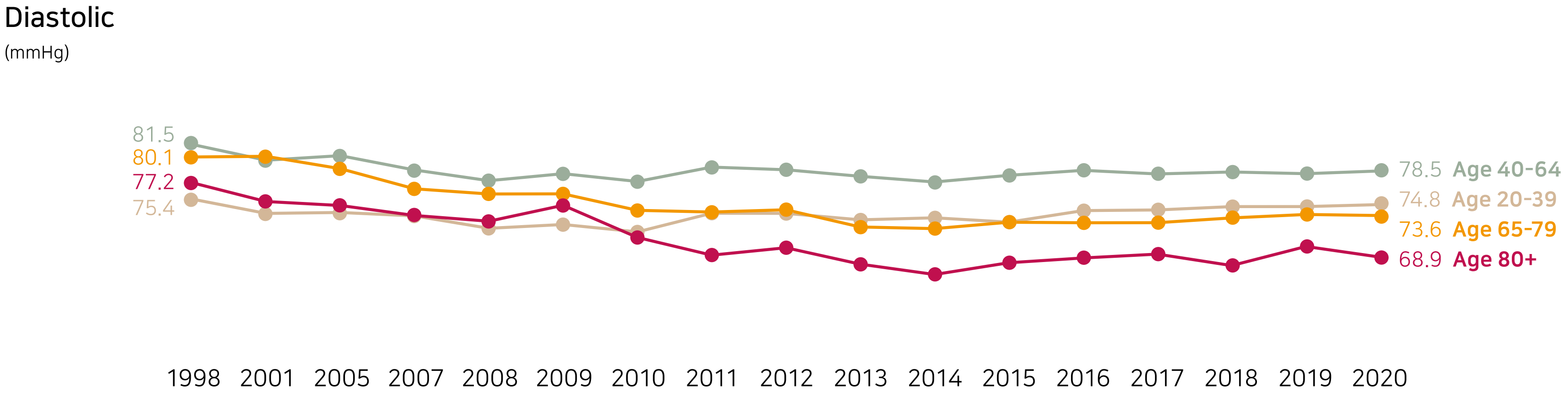

Data Source: Korea National Health and Nutrition Examination Survey 1998-2020

Trends of Hypertension Prevalence

(Age 20+, Age-standardized)

	Crude	Age-standardized
Age 20+	29 %	23 %

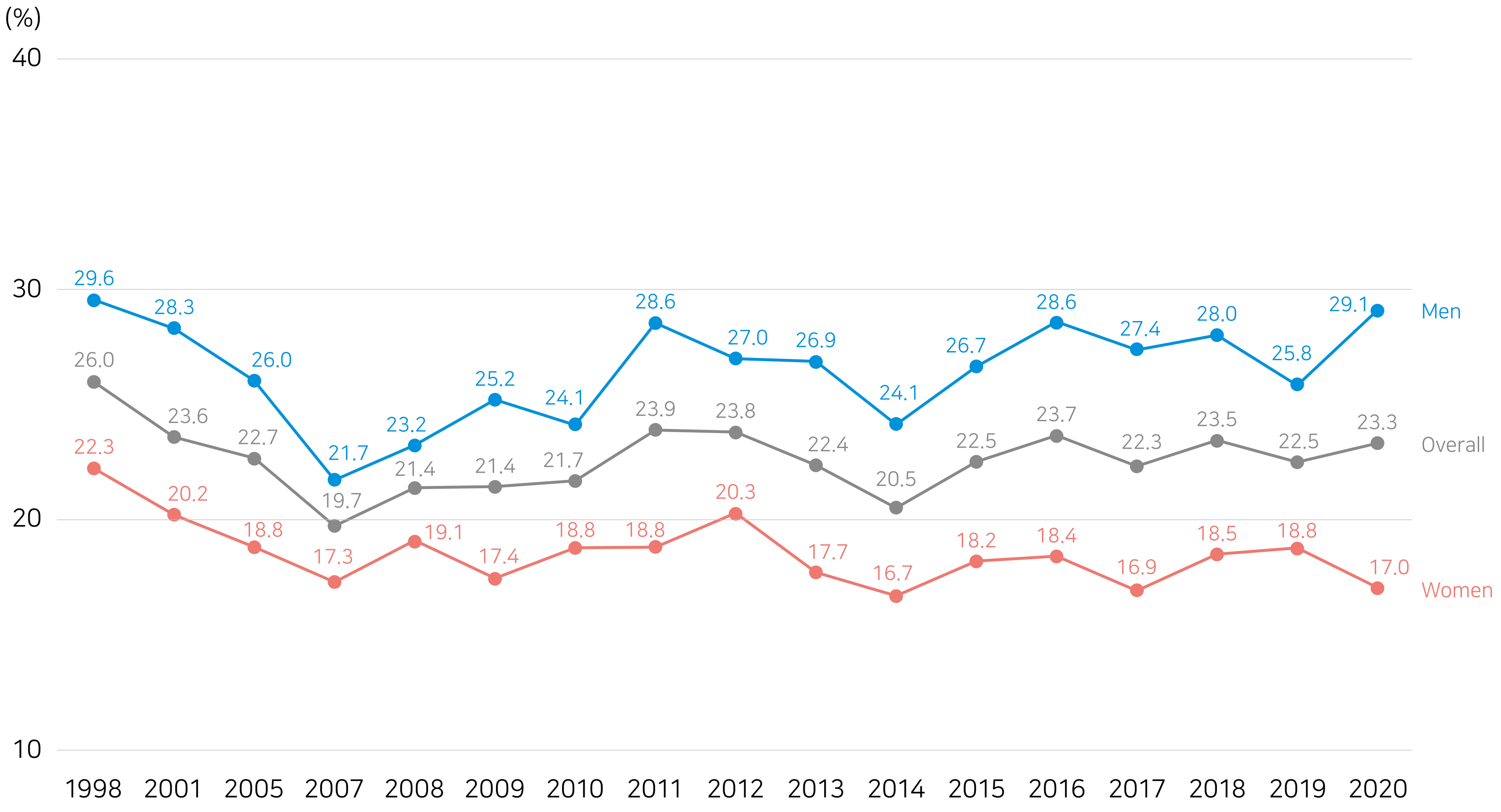

Data Source: Korea National Health and Nutrition Examination Survey 1998-2020
(Directly age-standardized to the 2005 projected population)

Trends of Hypertension Prevalence

(Age 30+, Age-standardized)

	Crude	Age-standardized
Age 30+	**34** %	**28** %

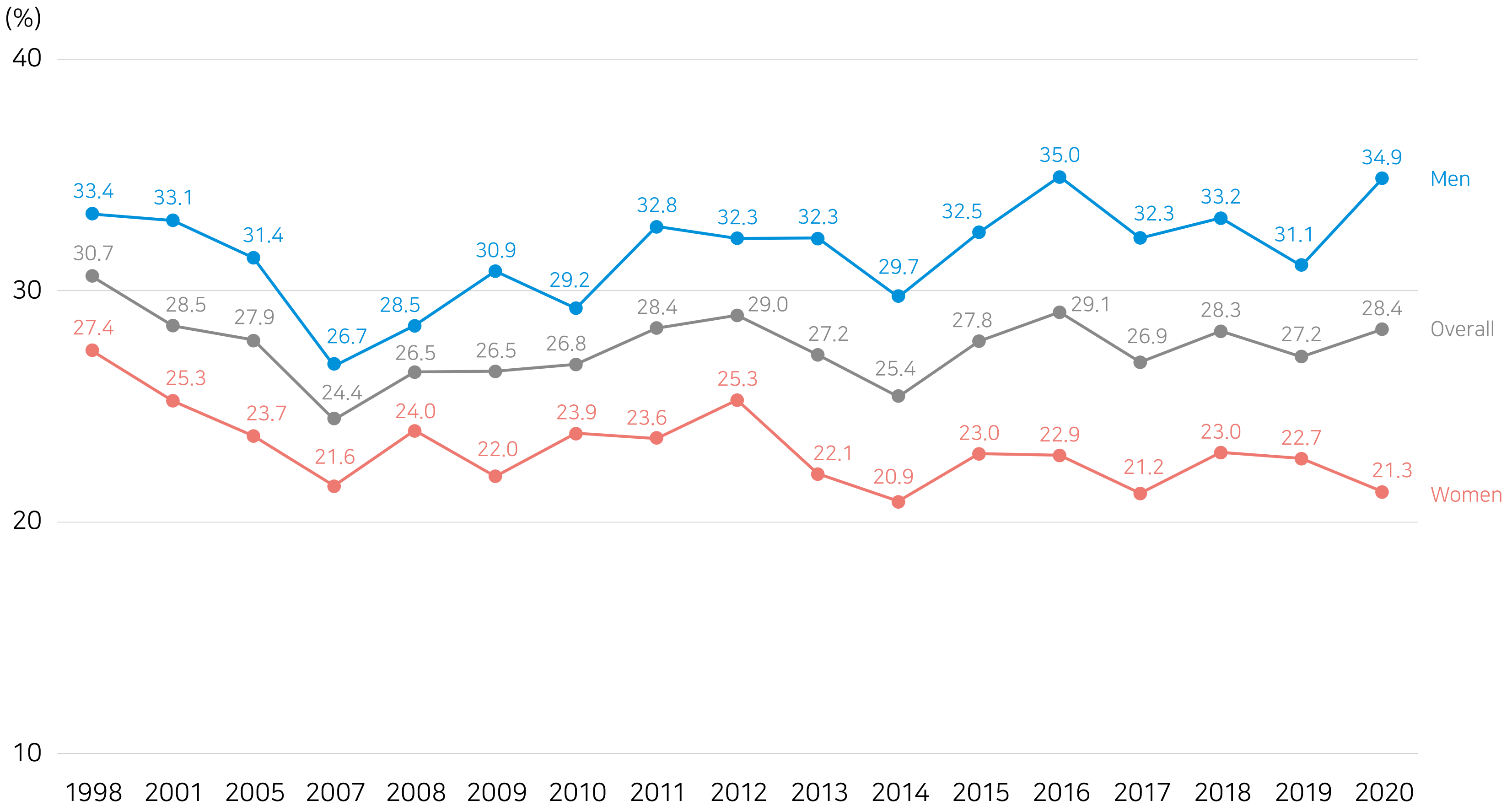

Data Source: Korea National Health and Nutrition Examination Survey 1998-2020
(Directly age-standardized to the 2005 projected population)

Trends of Hypertension Prevalence by Sex and Age

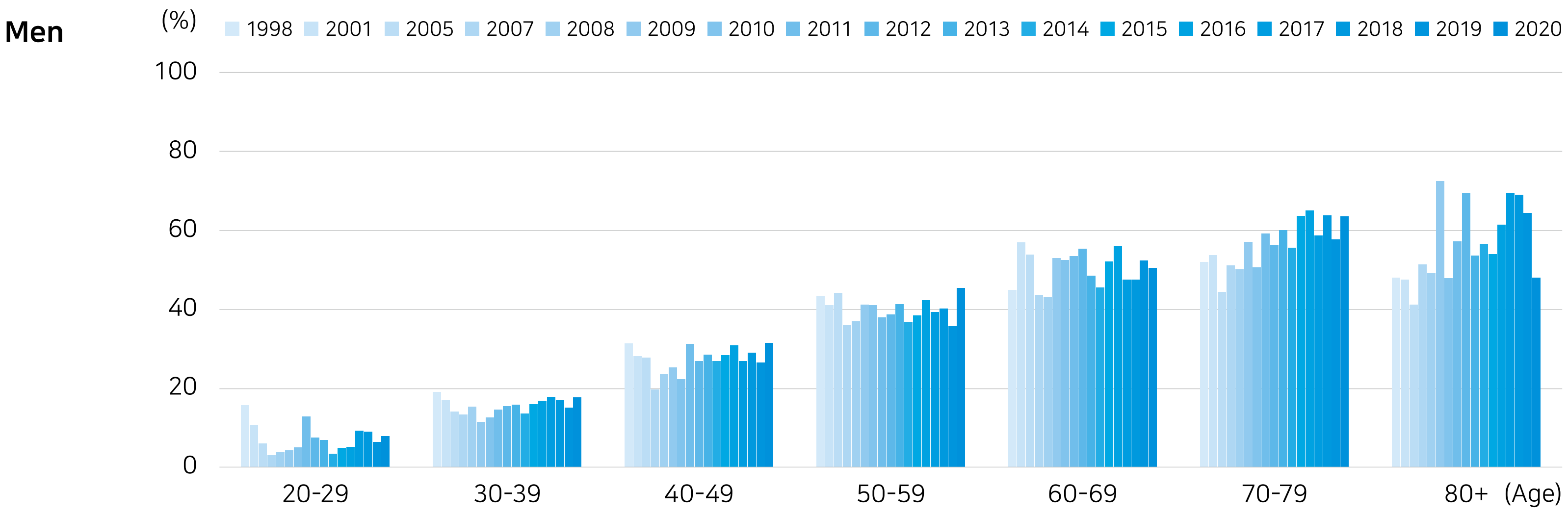

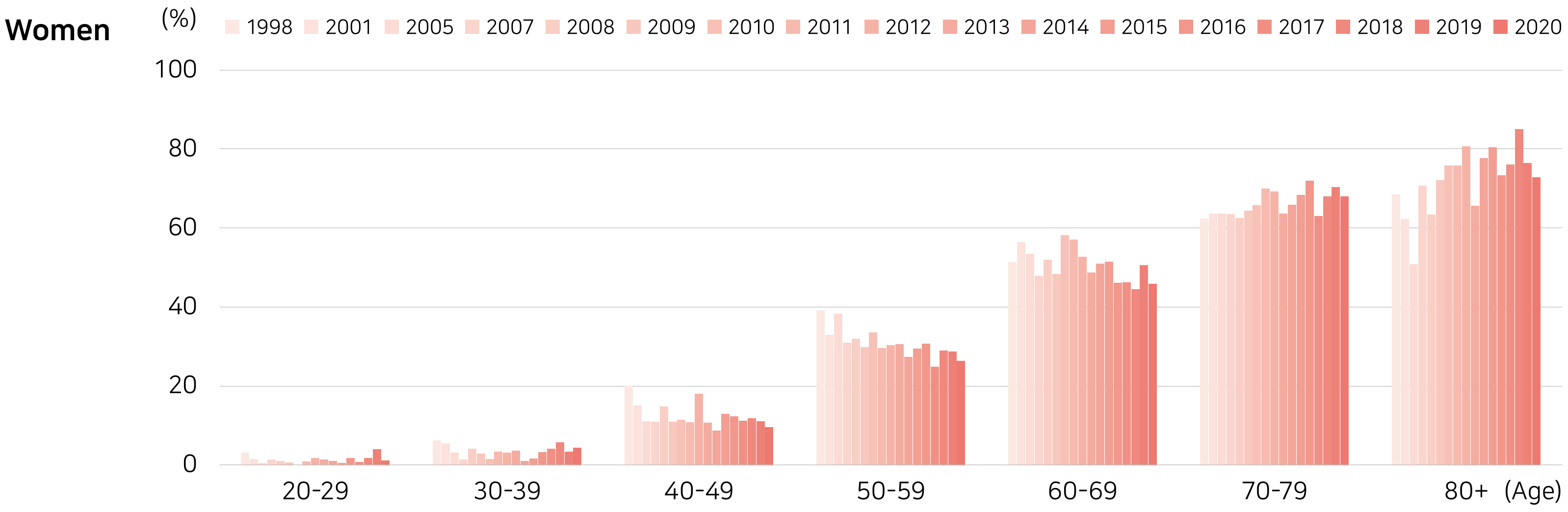

Data Source: Korea National Health and Nutrition Examination Survey 1998-2020

Trends of Number of People with Hypertension

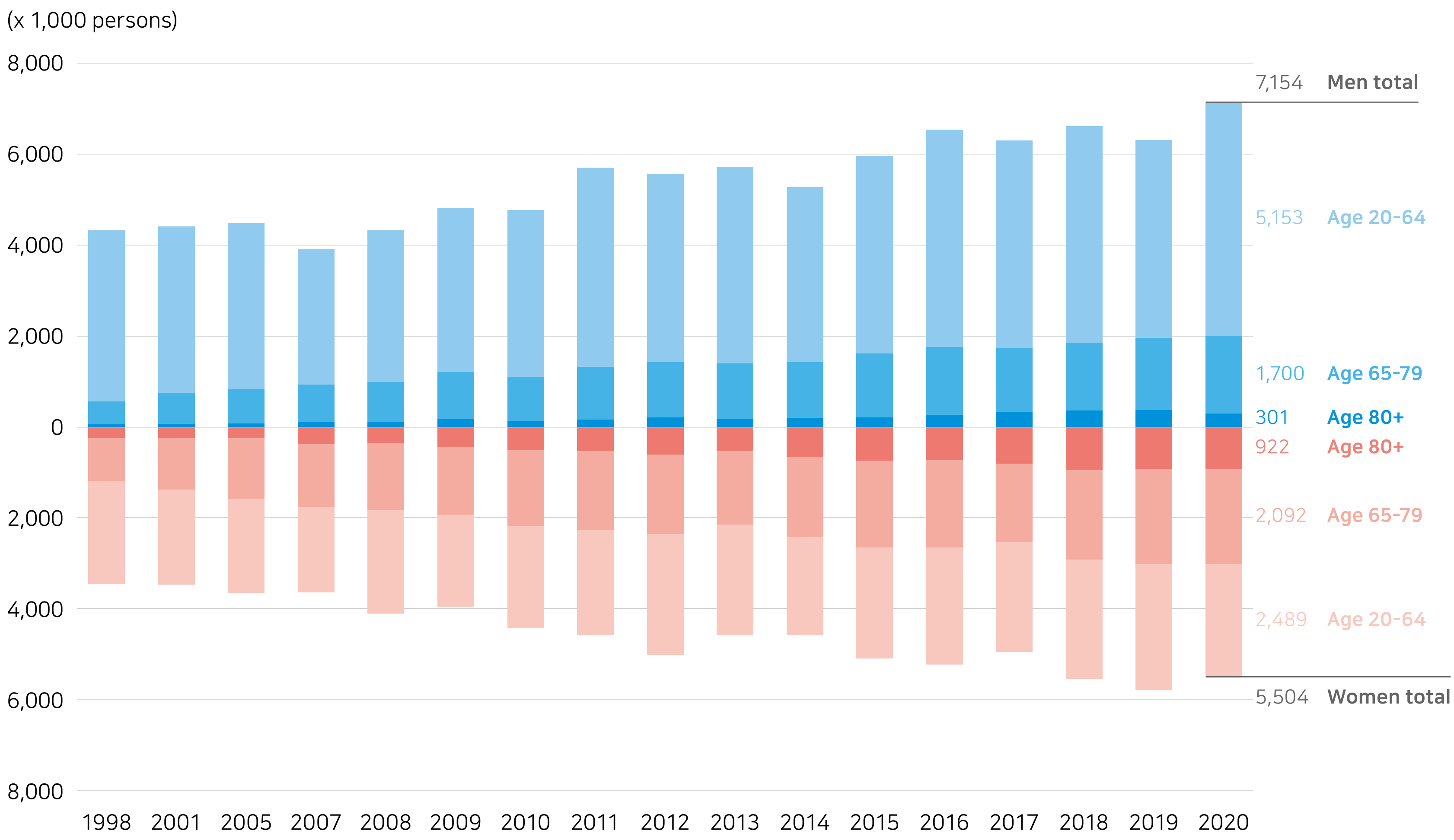

Data Source: Korea National Health and Nutrition Examination Survey 1998-2020

KOREA HYPERTENSION FACT SHEET 2022

Trends of Hypertension Management

Trends of Awareness Rate by Sex and Age

Trends of Treatment Rate by Sex and Age

Trends of Control Rate by Sex and Age

Trends of Awareness Rate by Sex and Age

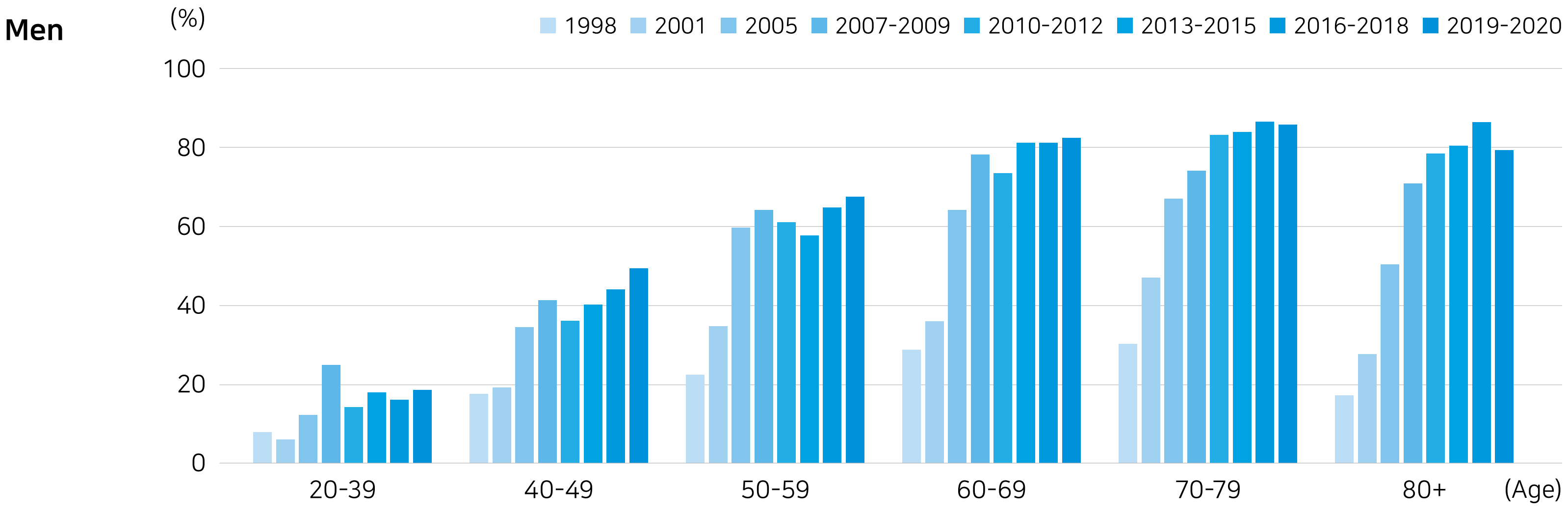

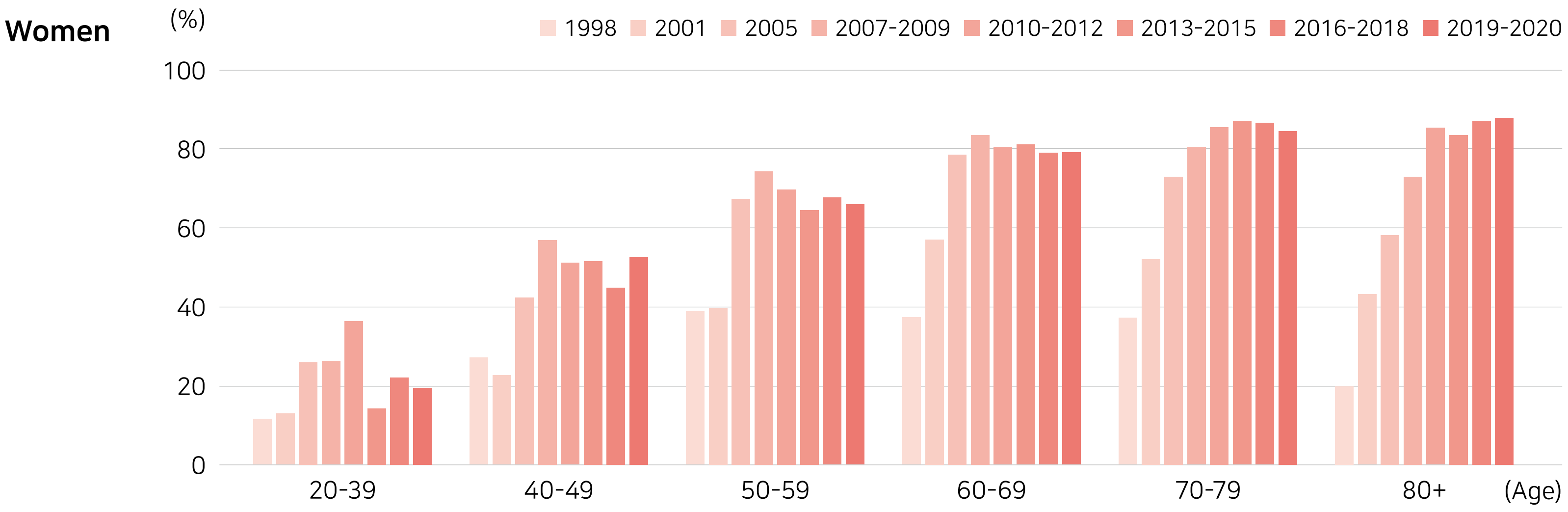

Data Source: Korea National Health and Nutrition Examination Survey 1998-2020

Trends of Treatment Rate by Sex and Age

Men

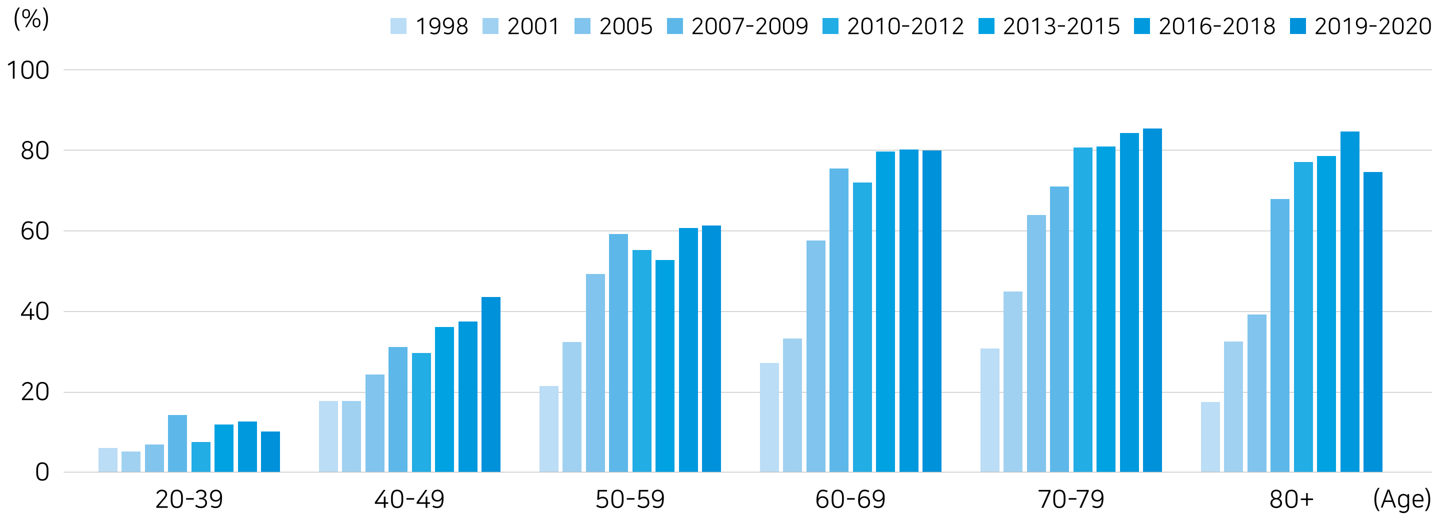

Women

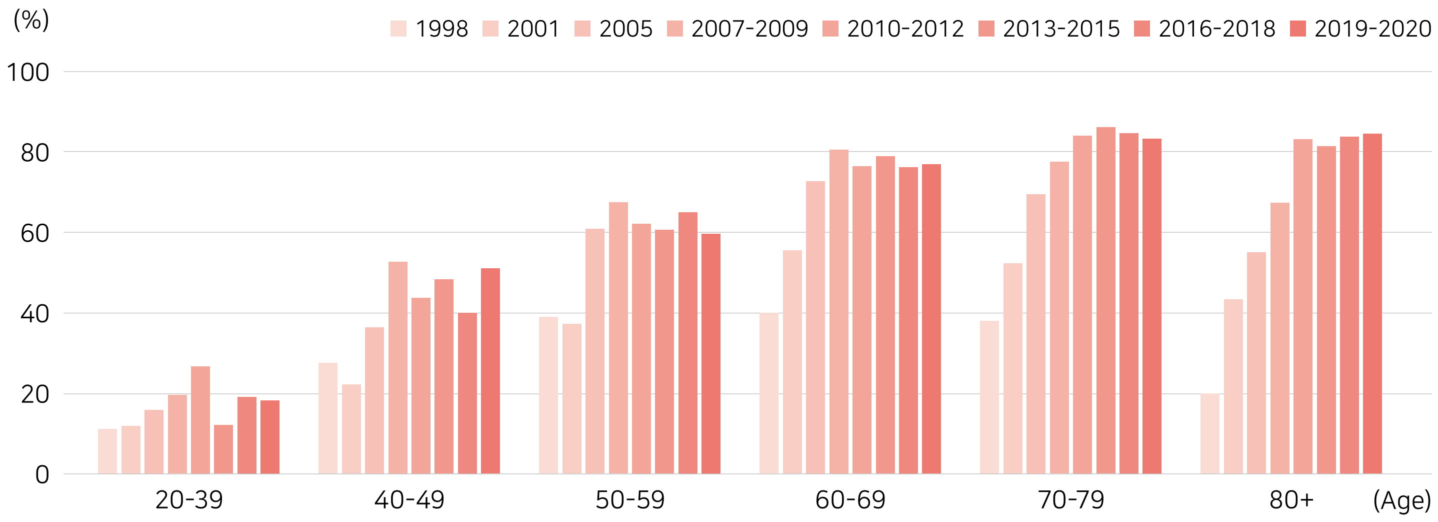

Data Source: Korea National Health and Nutrition Examination Survey 1998-2020

Trends of Control Rate by Sex and Age

(Among prevalent)

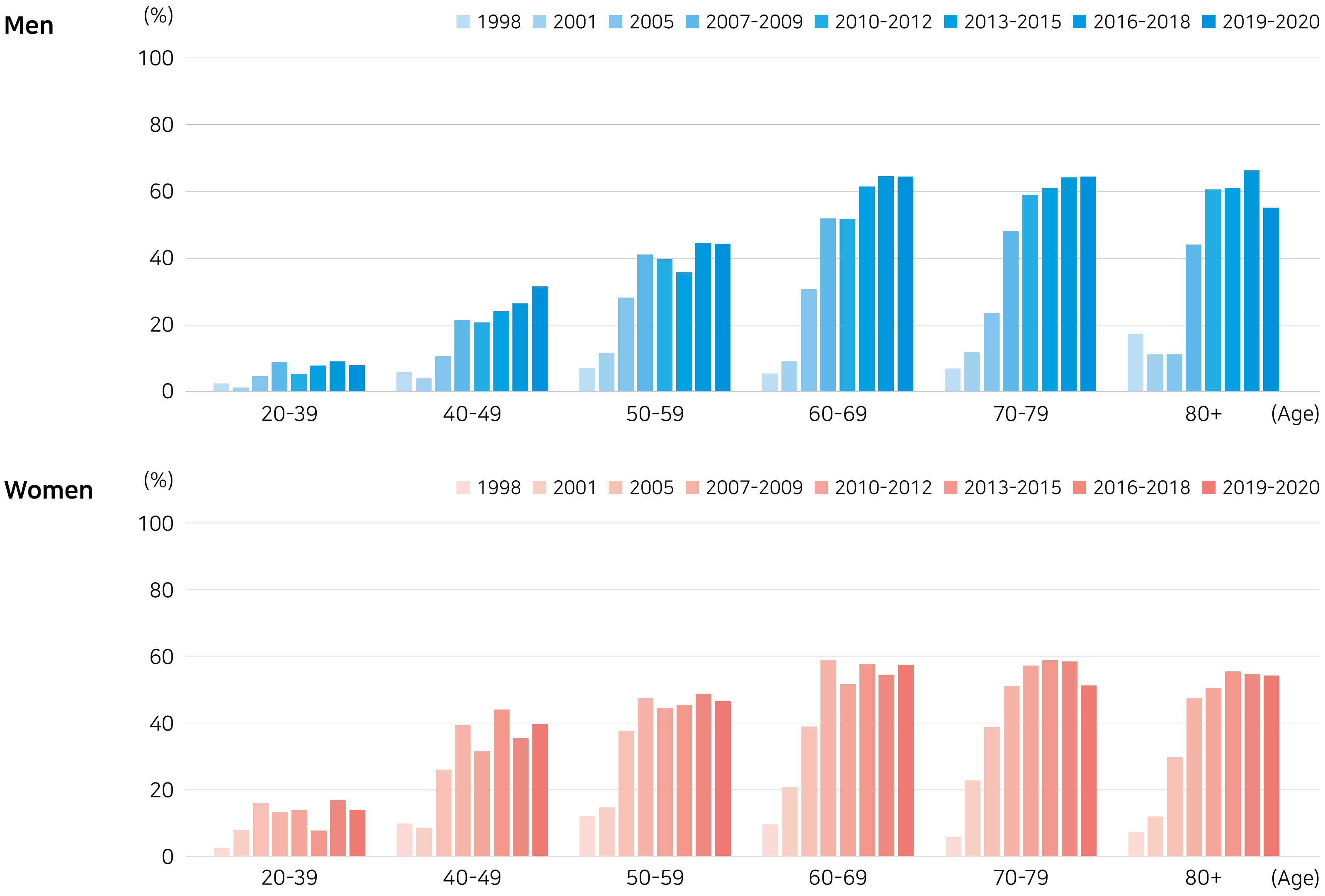

Data Source: Korea National Health and Nutrition Examination Survey 1998-2020

Trends of Control Rate by Sex and Age

(Among treated)

Men

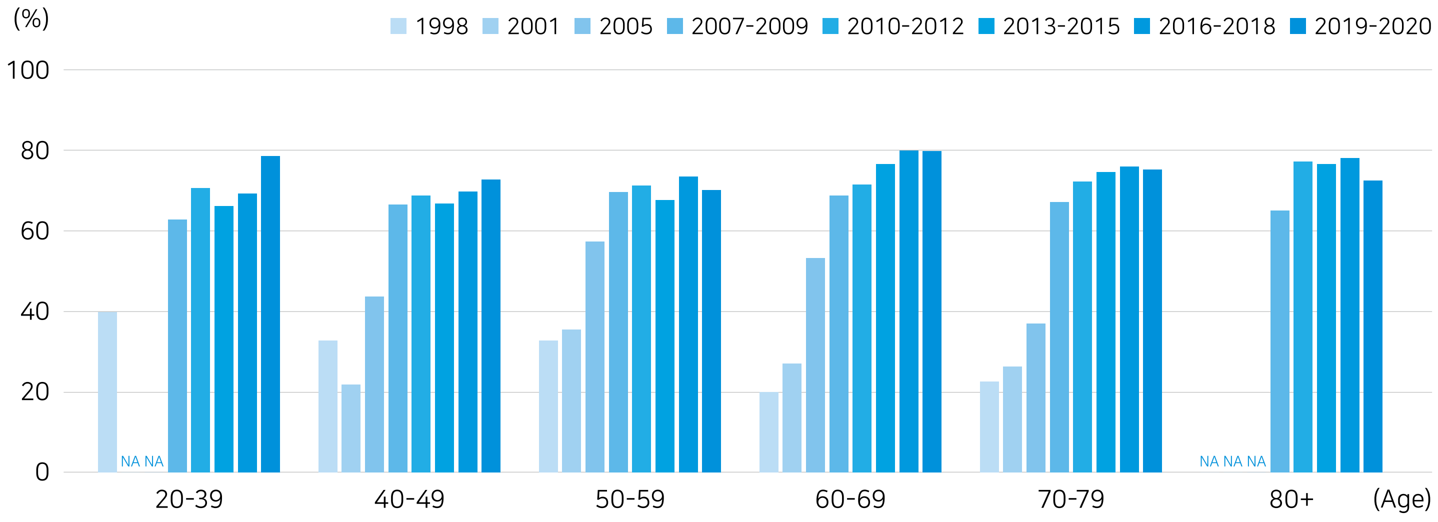

Women

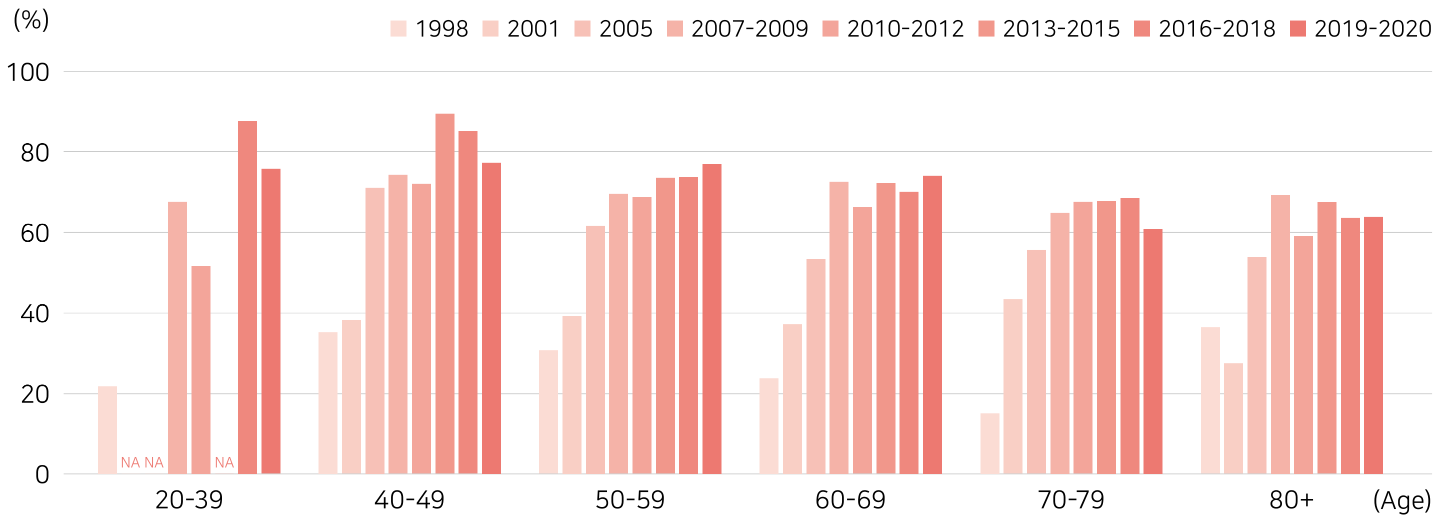

NA = Denominator less than 10

Data Source: Korea National Health and Nutrition Examination Survey 1998-2020

KOREA HYPERTENSION FACT SHEET 2022

Healthcare Utilization for Hypertension

Trends of Healthcare Utilization for Hypertension
Trends of Co-Treatment for Dyslipidemia and Diabetes
Trends of Antihypertensive Medication Use
Composition of Antihypertensive Treatment
Antihypertensive Medication Use by Sex and Age

Trends of Healthcare Utilization for Hypertension

(Age 20+)

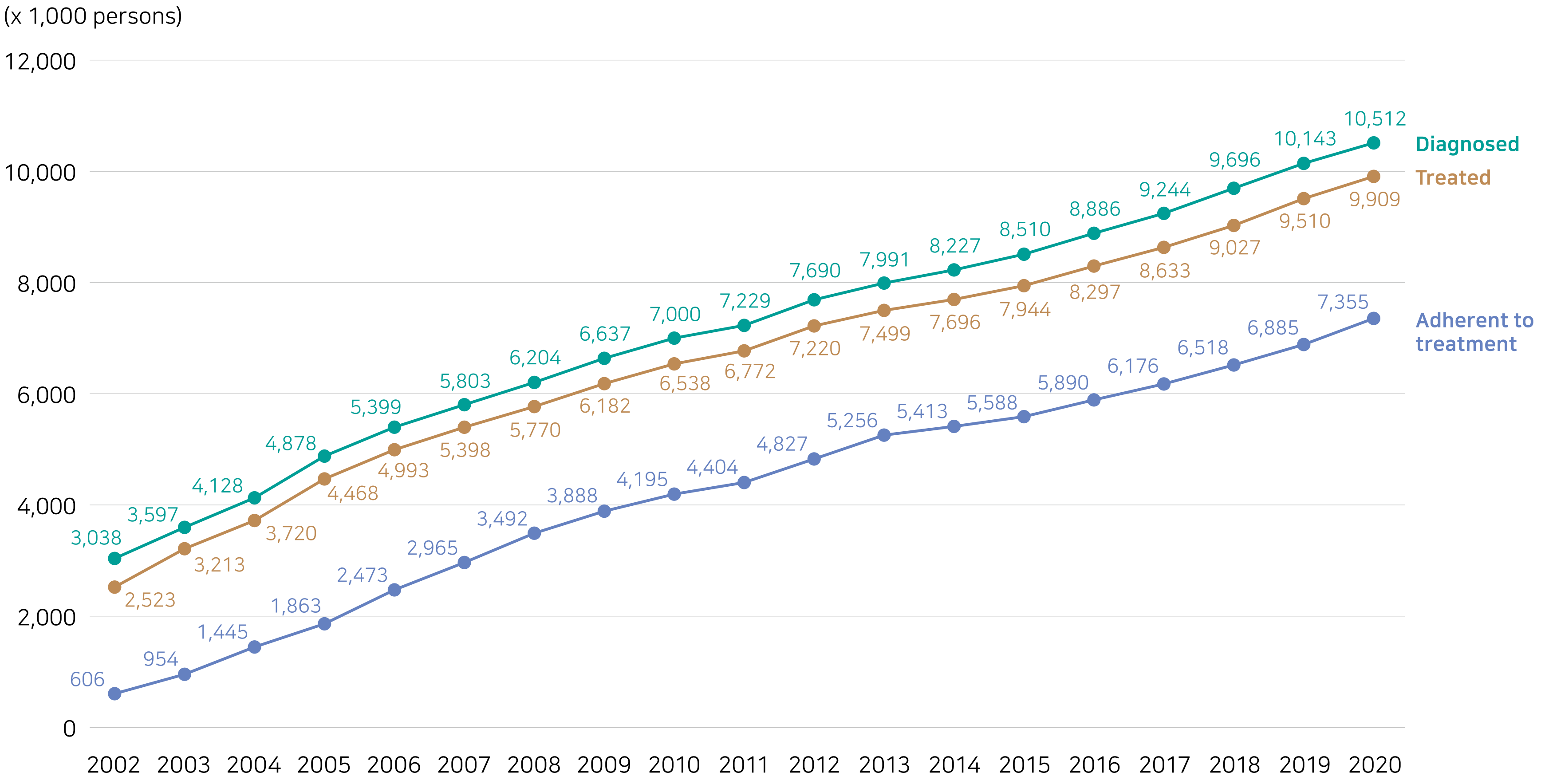

Data Source: Korea National Health Insurance Big Data 2002-2020

Trends of Co-Treatment for Dyslipidemia and Diabetes

(Age 20+)

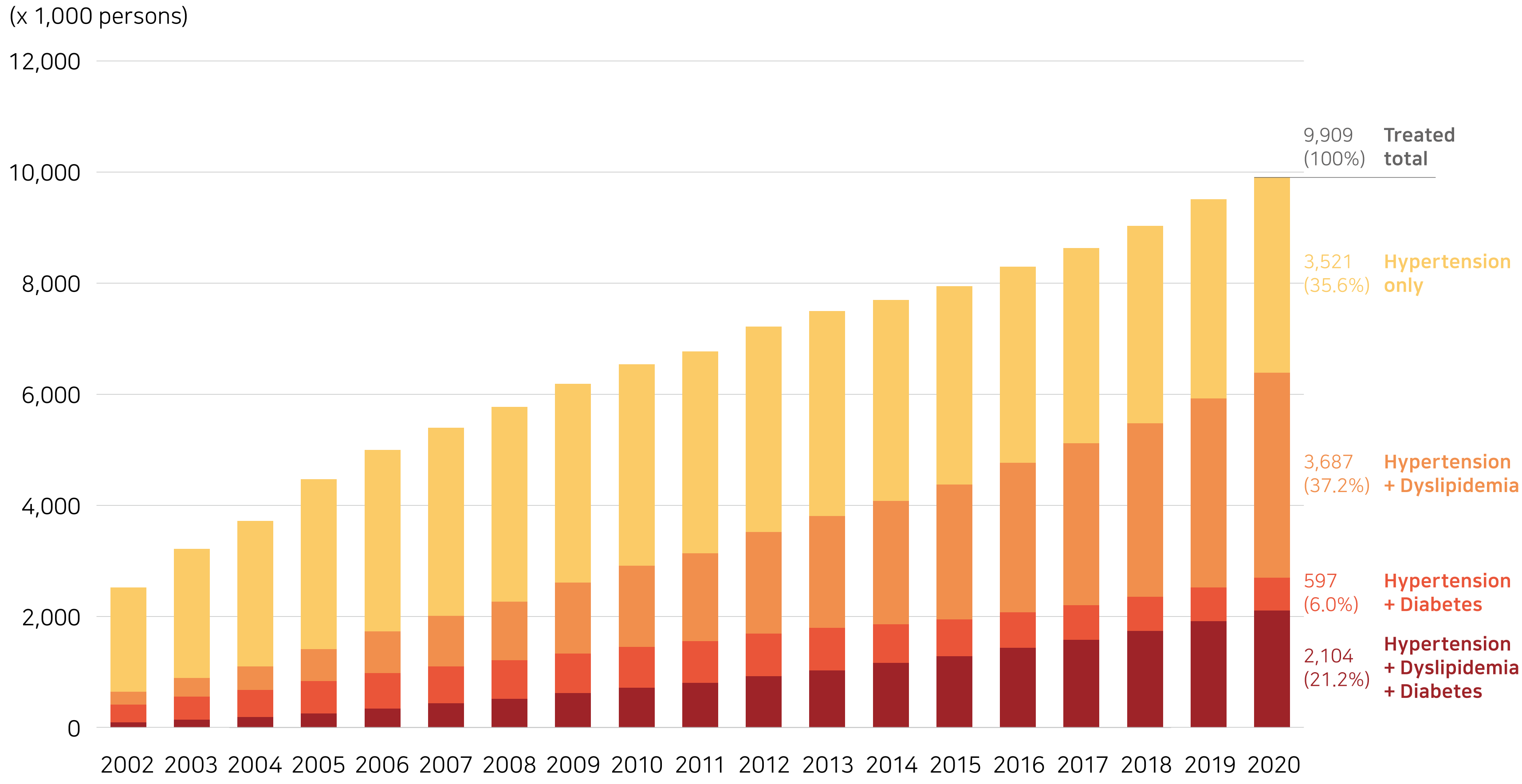

Data Source: Korea National Health Insurance Big Data 2002-2020

Trends of Antihypertensive Medication Use

(Age 20+)

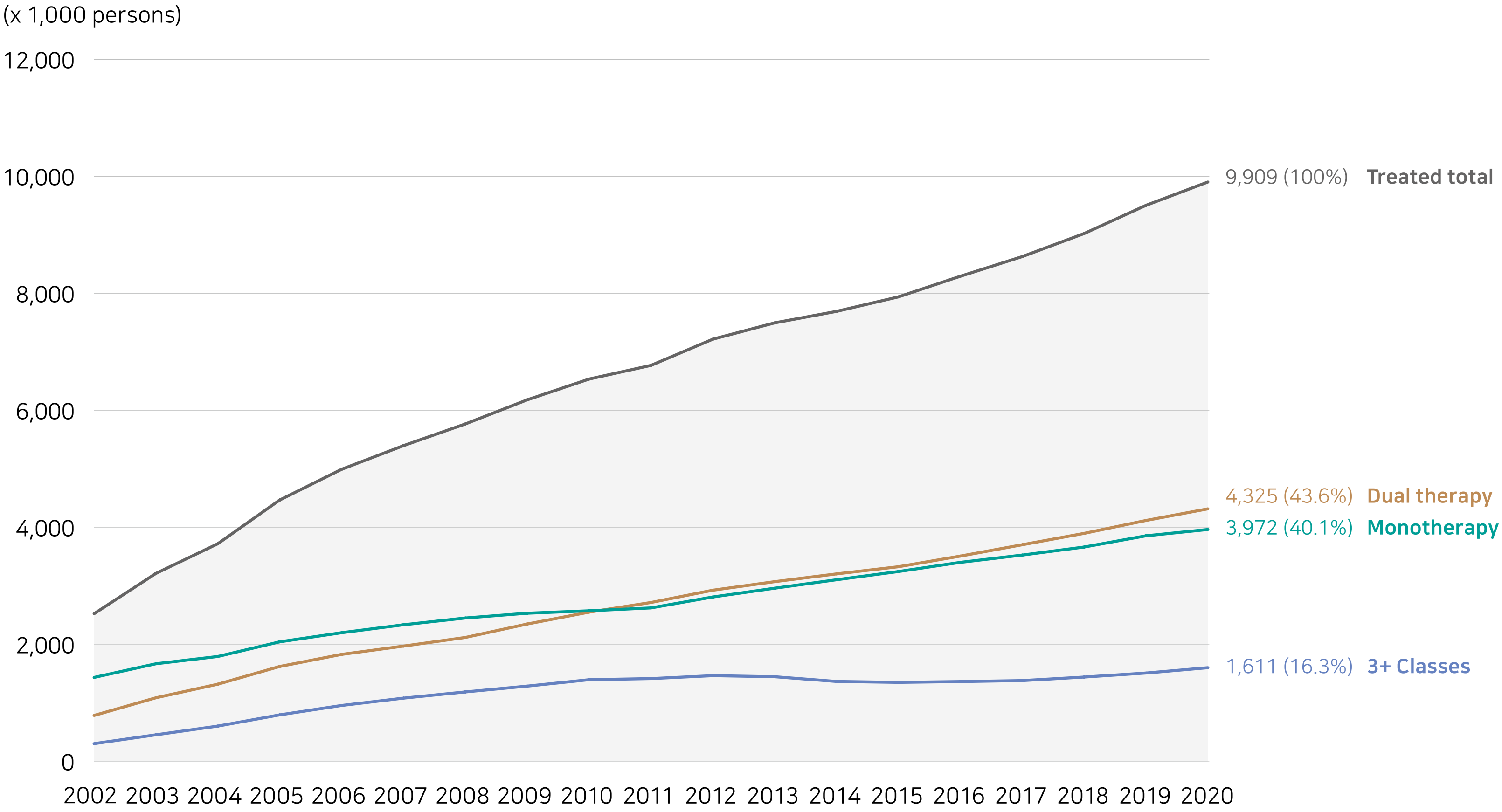

Data Source: Korea National Health Insurance Big Data 2002-2020

Trends of Antihypertensive Medication Use

(Age 20+)

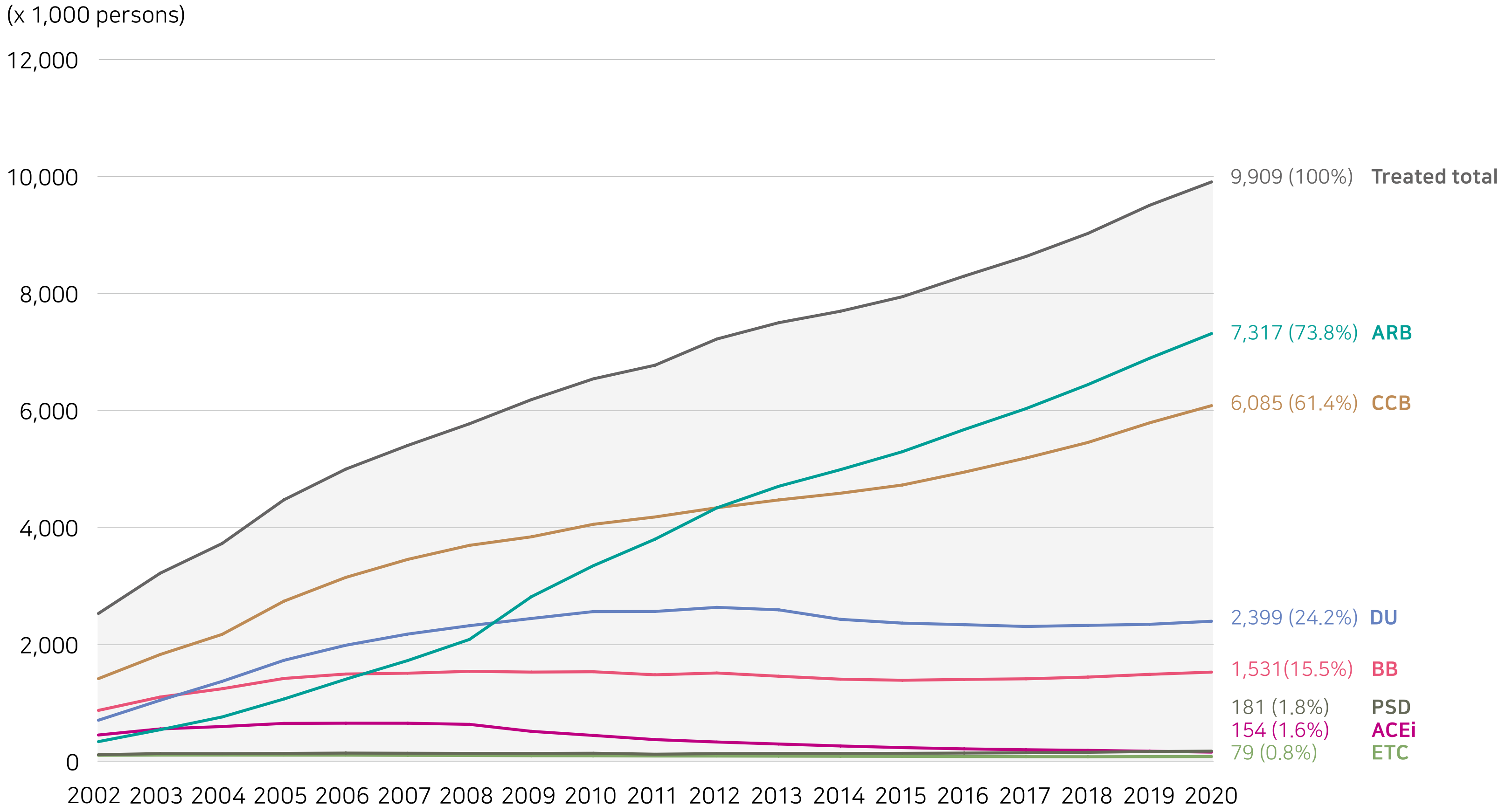

Data Source: Korea National Health Insurance Big Data 2002-2020

Composition of Antihypertensive Treatment

(Age 20+)

Monotherapy

Among 4.0 million, %

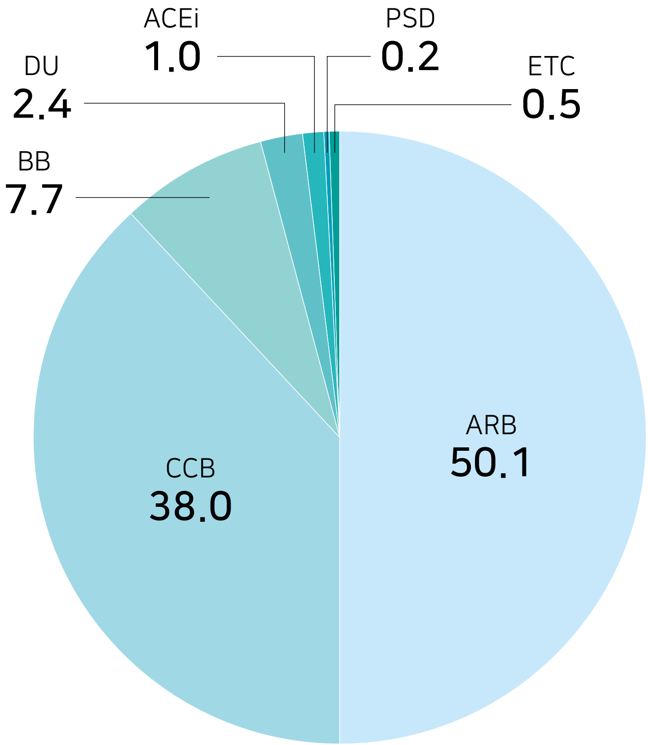

Dual therapy

Among 4.3 million, %

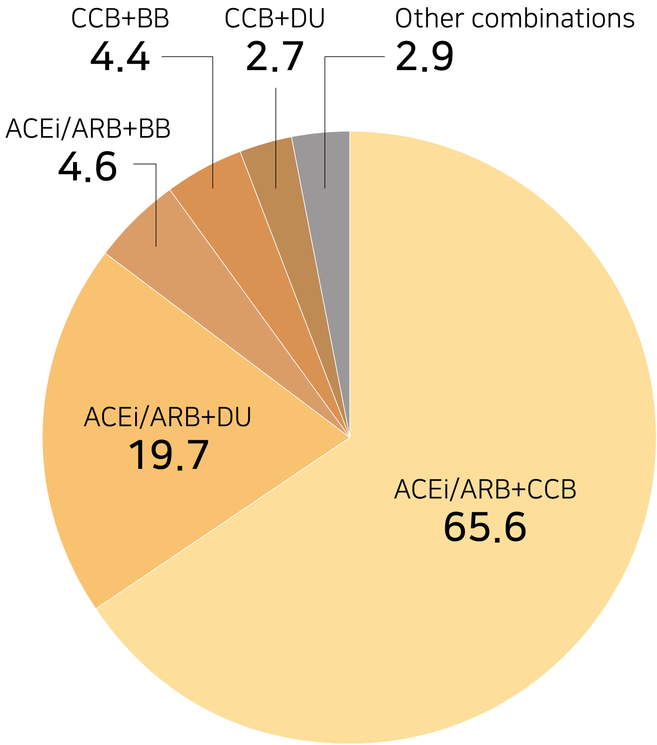

Data Source: Korea National Health Insurance Big Data 2020

Composition of Antihypertensive Treatment

(Age 20+)

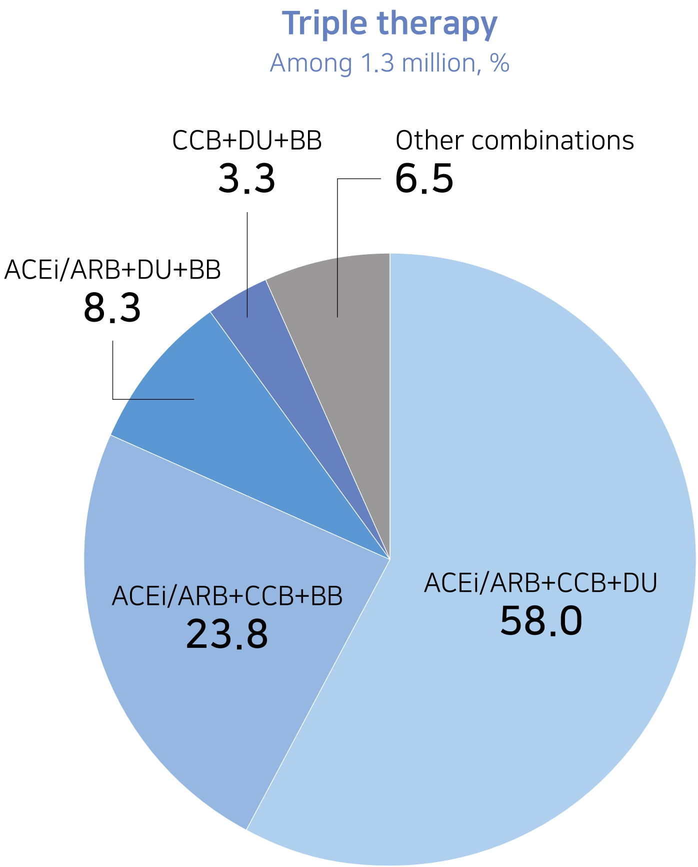

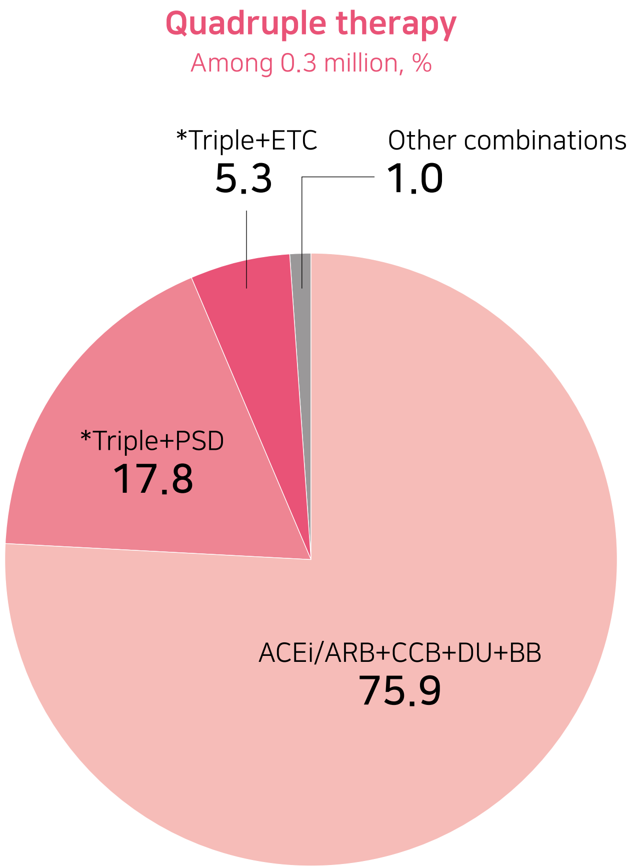

Data Source: Korea National Health Insurance Big Data 2020

*Triple = Three of the ACEi/ARB, CCB, DU, or BB

Antihypertensive Medication Use by Sex and Age

(Among treated, %)

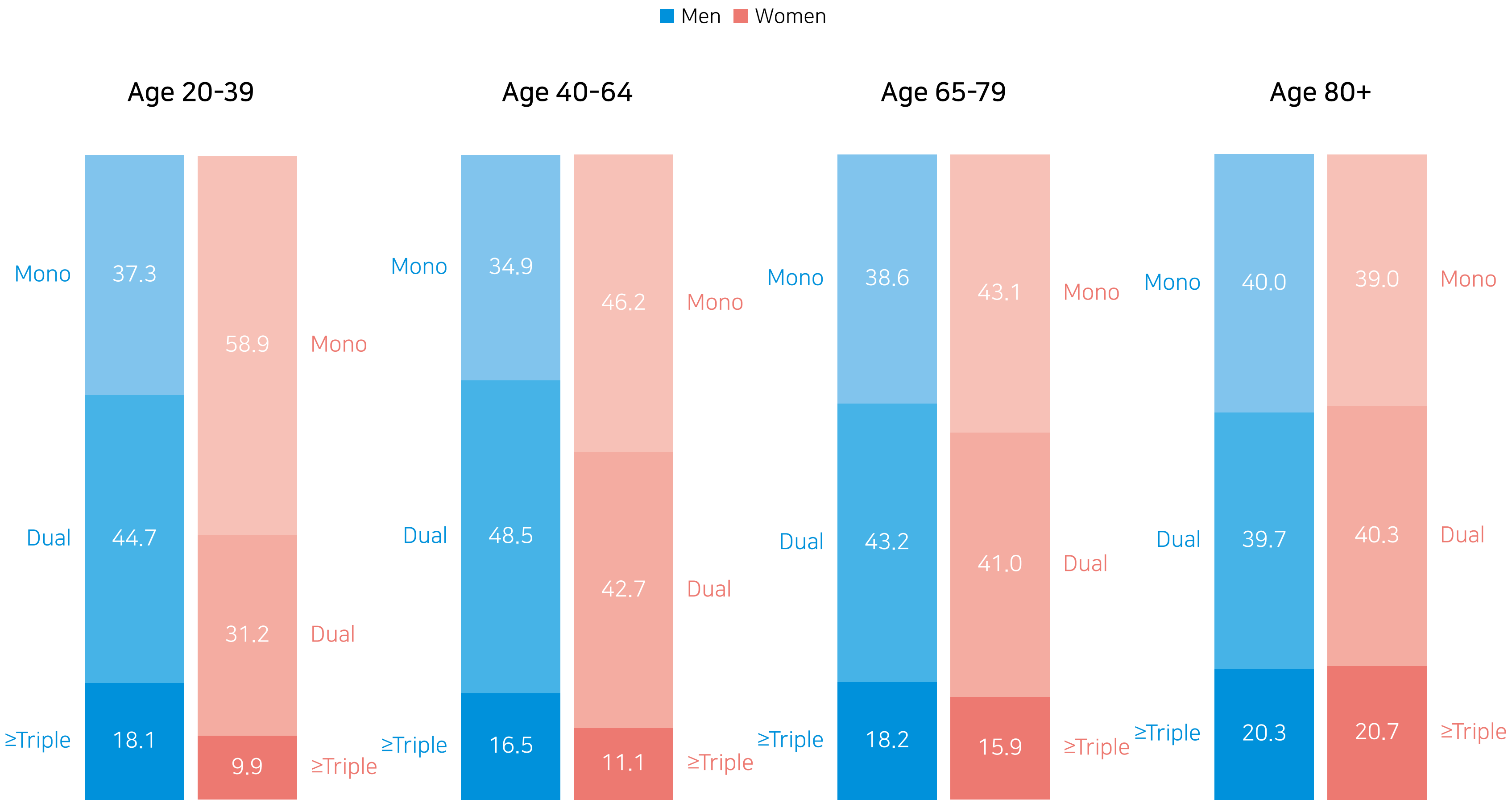

Data Source: Korea National Health Insurance Big Data 2020

Antihypertensive Medication Use by Sex and Age

(Among treated, %)

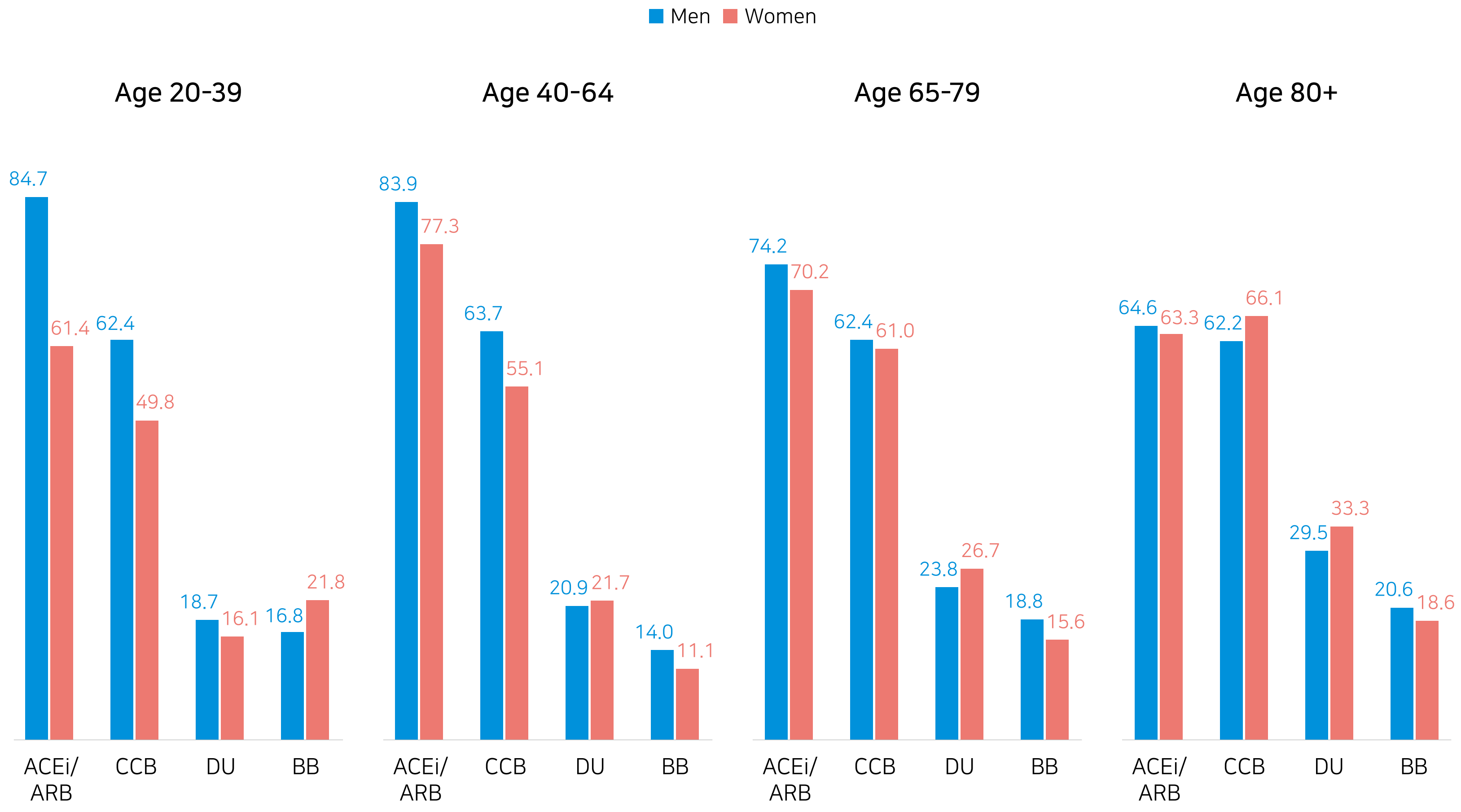

Data Source: Korea National Health Insurance Big Data 2020

Hypertension in the Elderly

Trends of Number of People with Hypertension by Management Status

Trends of Awareness Rate in the Elderly

Trends of Treatment Rate in the Elderly

Trends of Adherence Rate in the Elderly

Trends of Combination Therapy Rate in the Elderly

Trends of Average Blood Pressure in the Elderly

Trends of Number of People with Hypertension by Management Status

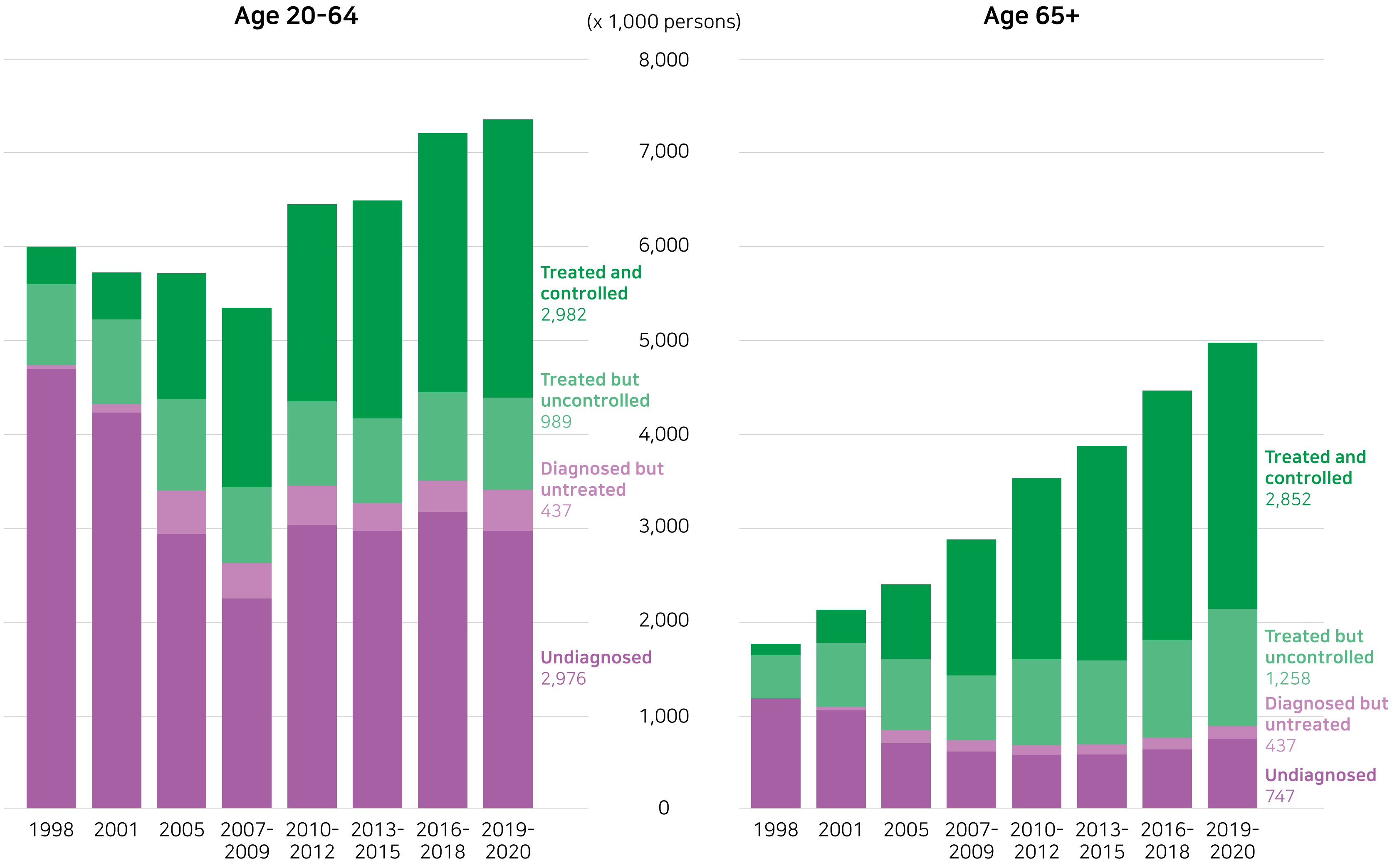

Data Source: Korea National Health and Nutrition Examination Survey 1998-2020

Trends of Awareness Rate in the Elderly

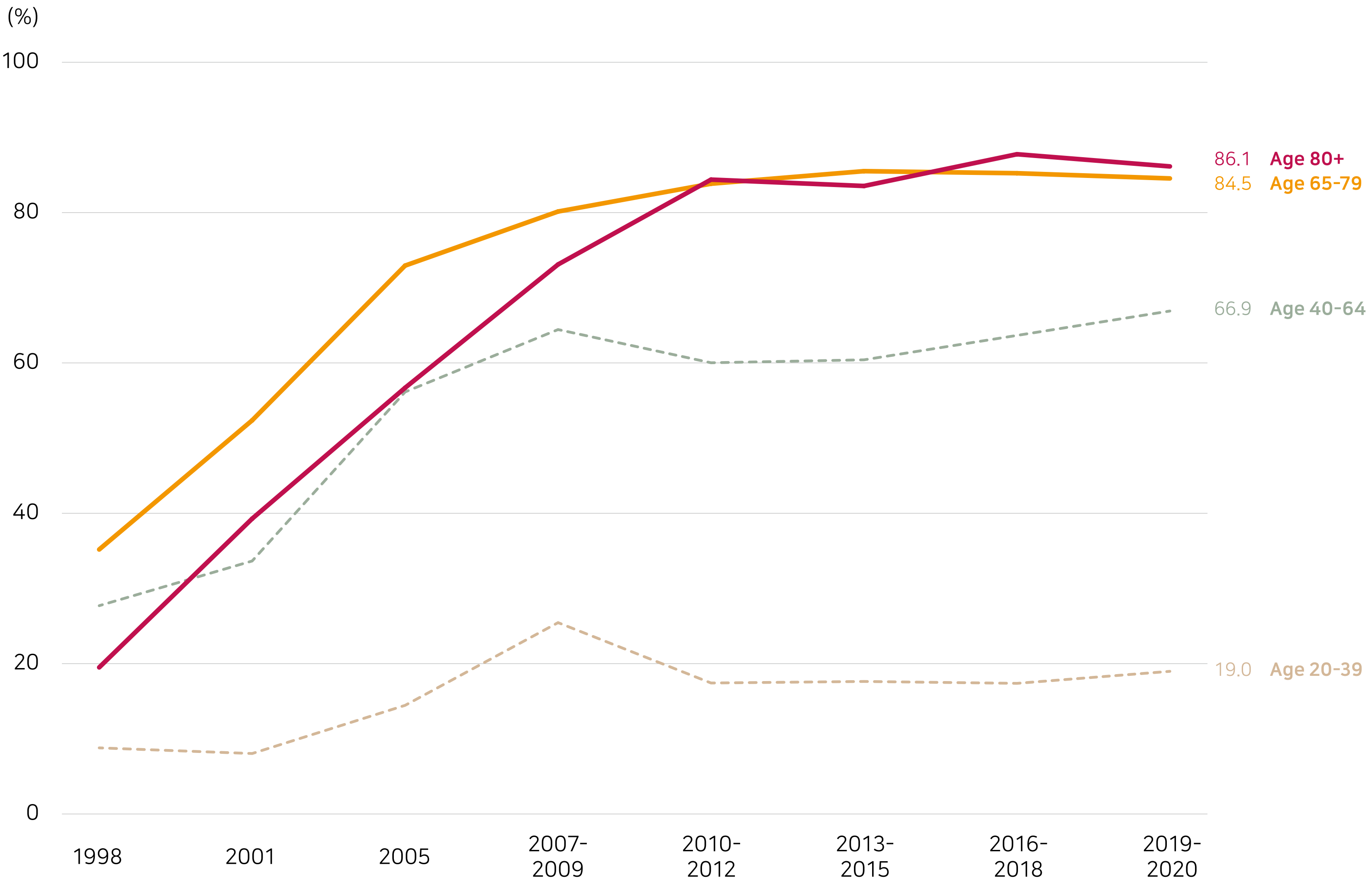

Data Source: Korea National Health and Nutrition Examination Survey 1998-2020

Trends of Treatment Rate in the Elderly

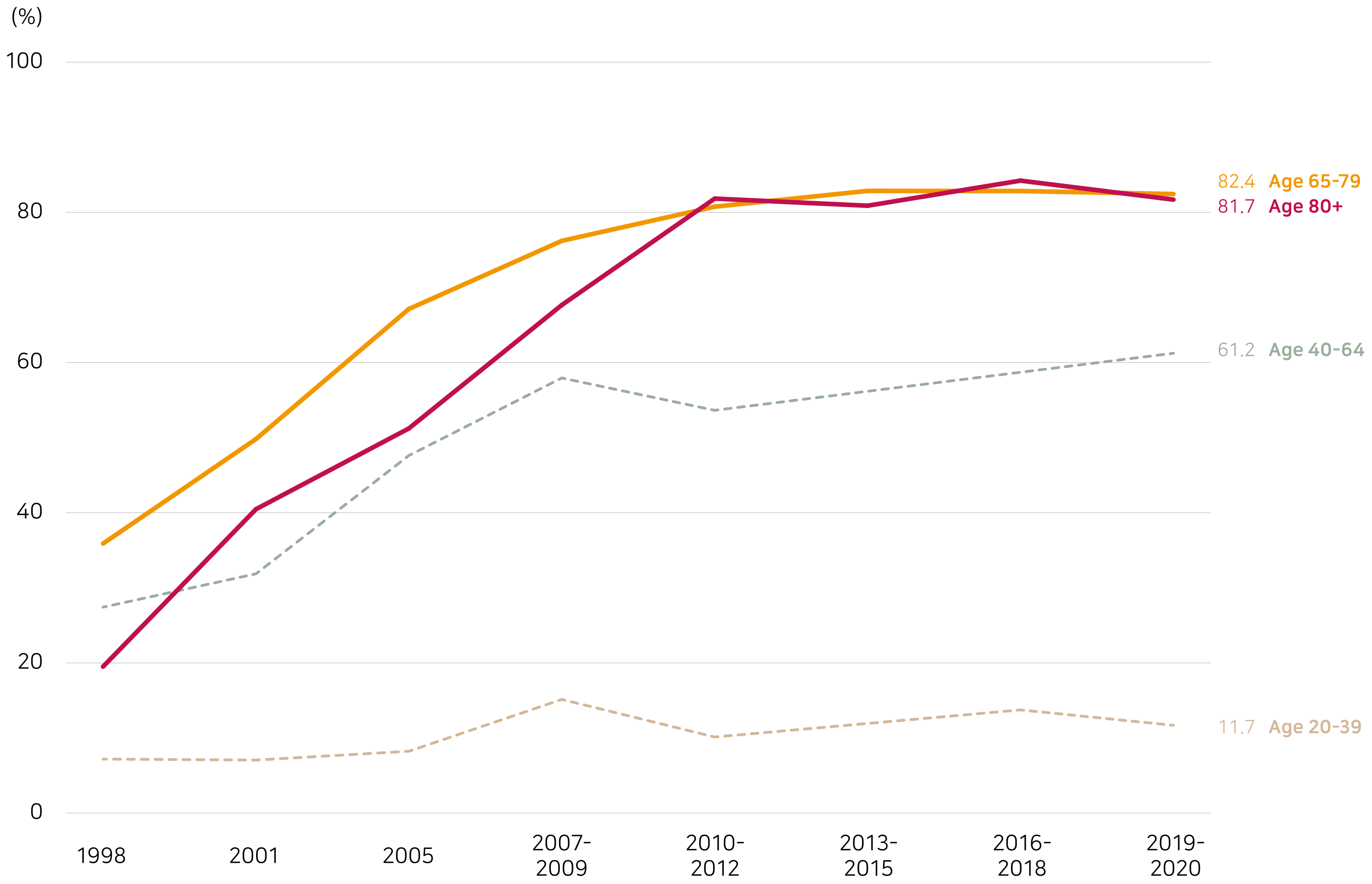

Data Source: Korea National Health and Nutrition Examination Survey 1998-2020

Trends of Adherence Rate in the Elderly

(Among treated)

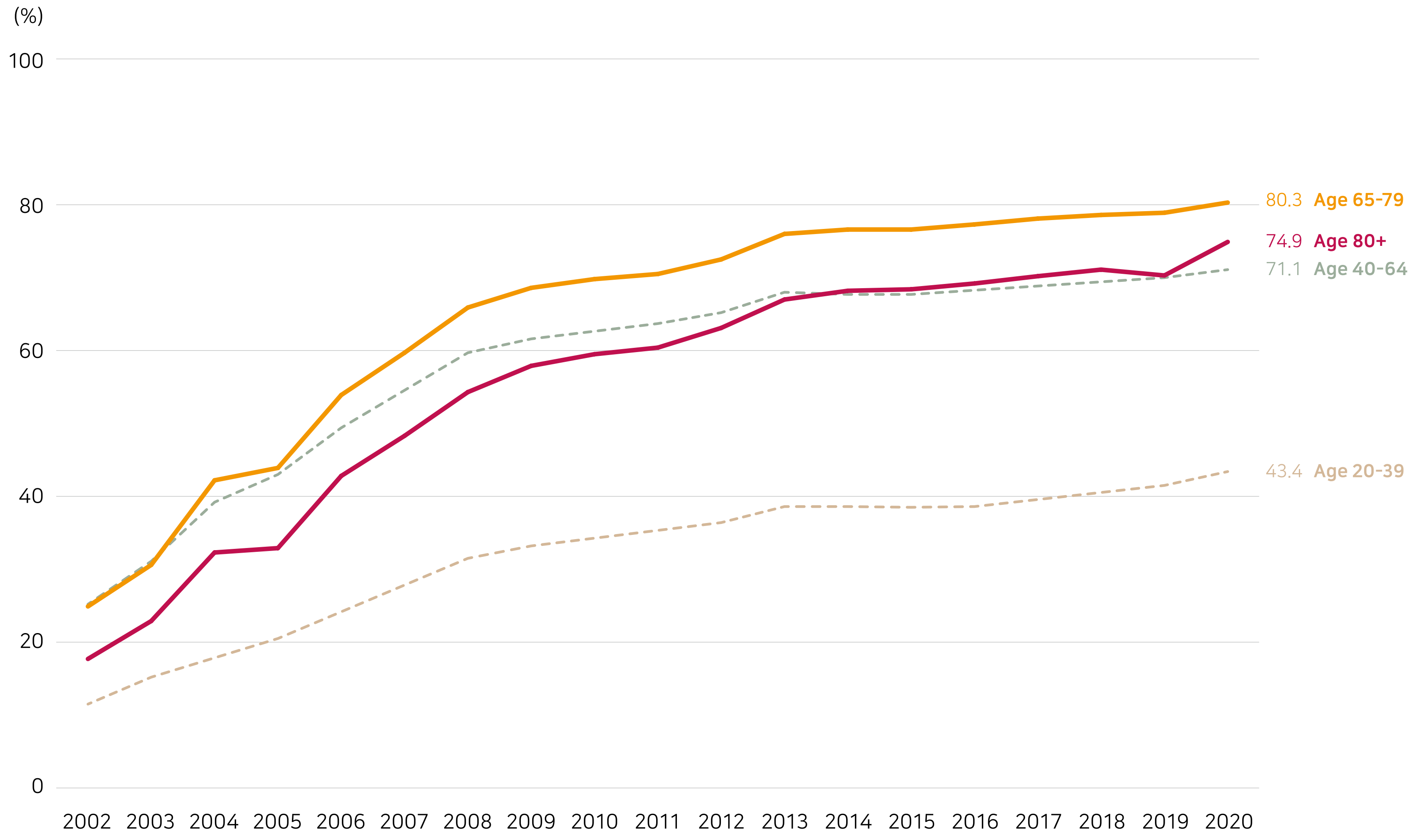

Data Source: Korea National Health Insurance Big Data 2002-2020

Trends of Combination Therapy Rate in the Elderly

(Among treated)

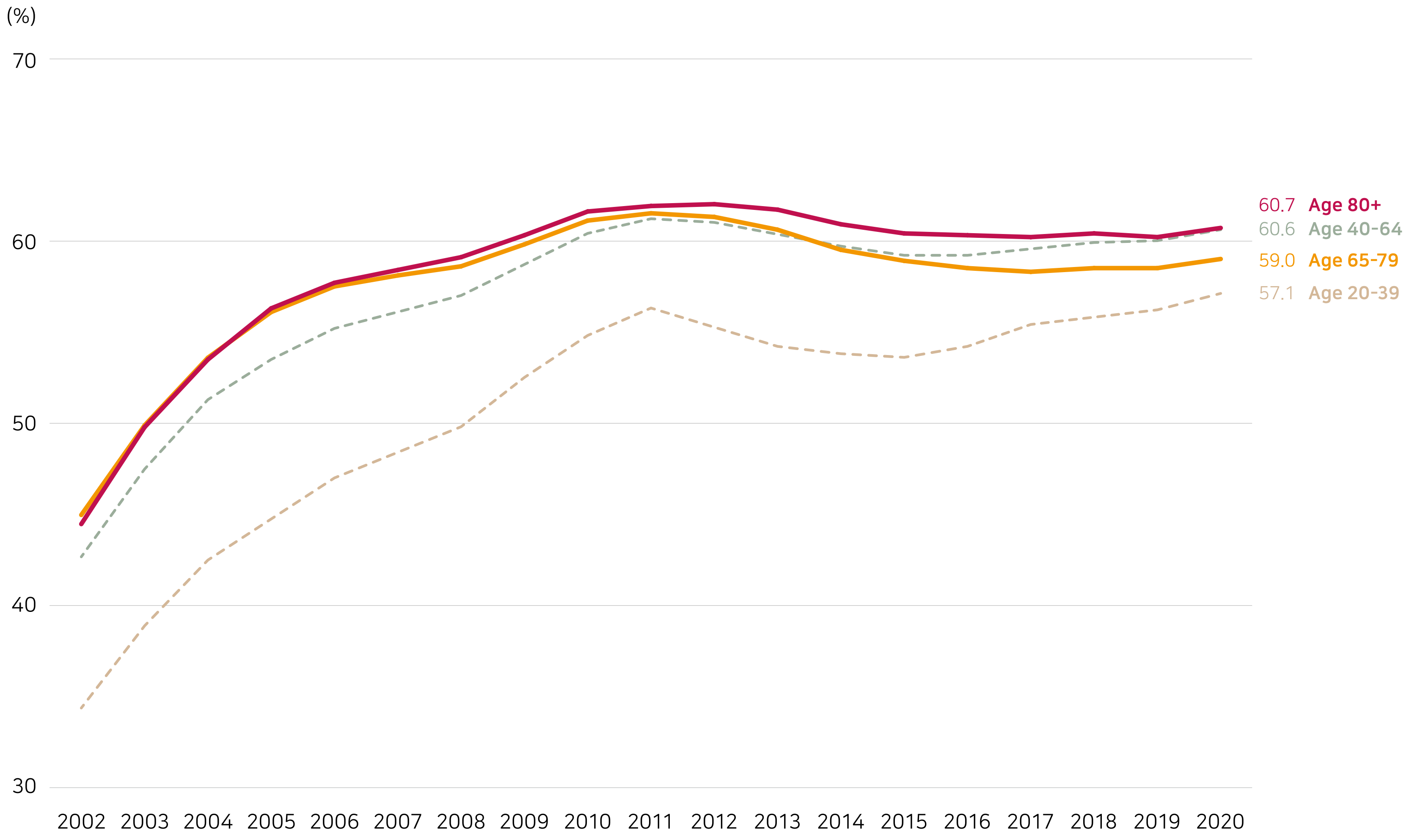

Data Source: Korea National Health Insurance Big Data 2002-2020

Trends of Average Blood Pressure in the Elderly

(Among prevalent)

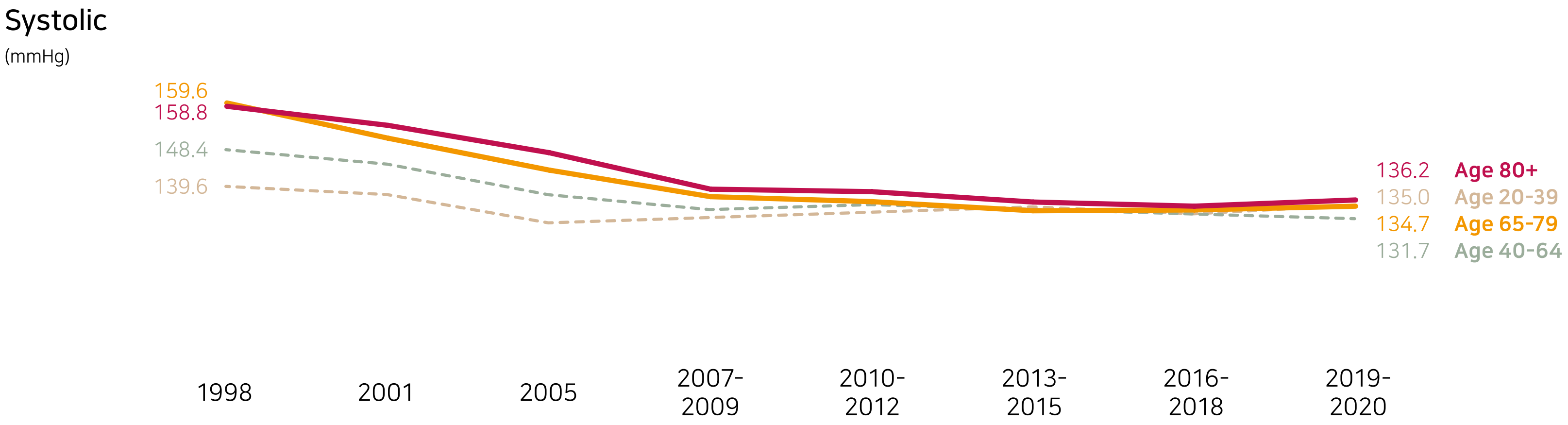

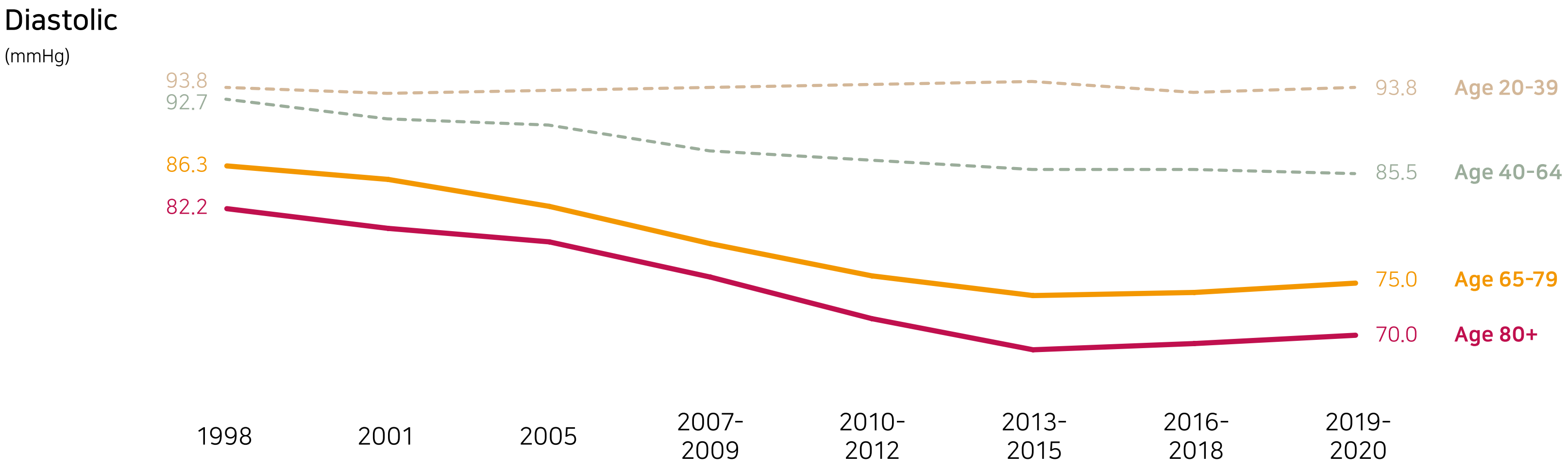

Data Source: Korea National Health and Nutrition Examination Survey 1998-2020

Trends of Average Blood Pressure in the Elderly

(Among treated)

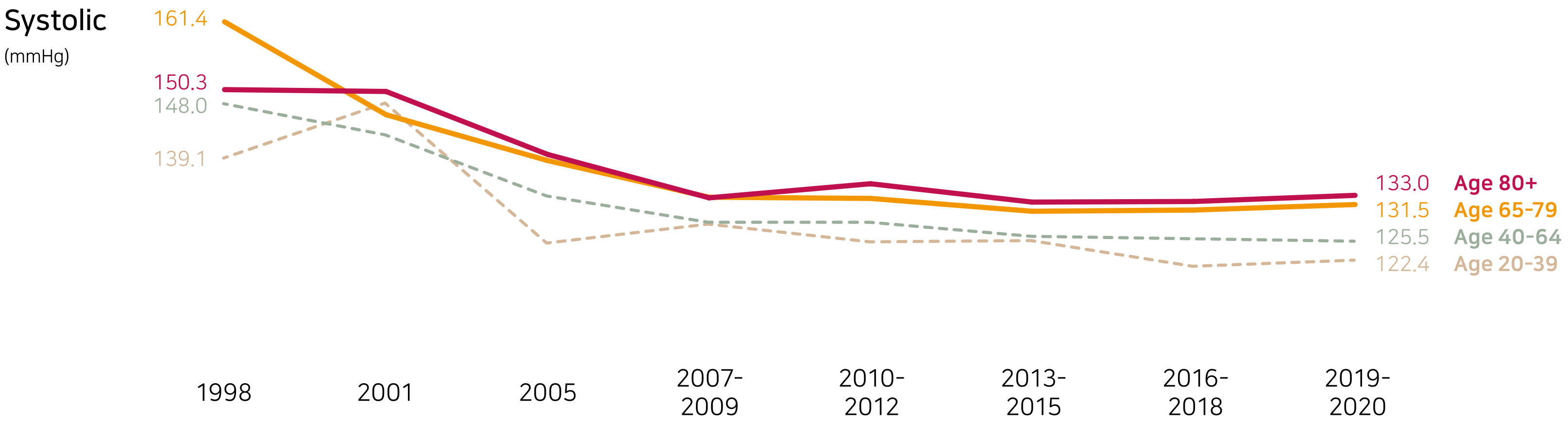

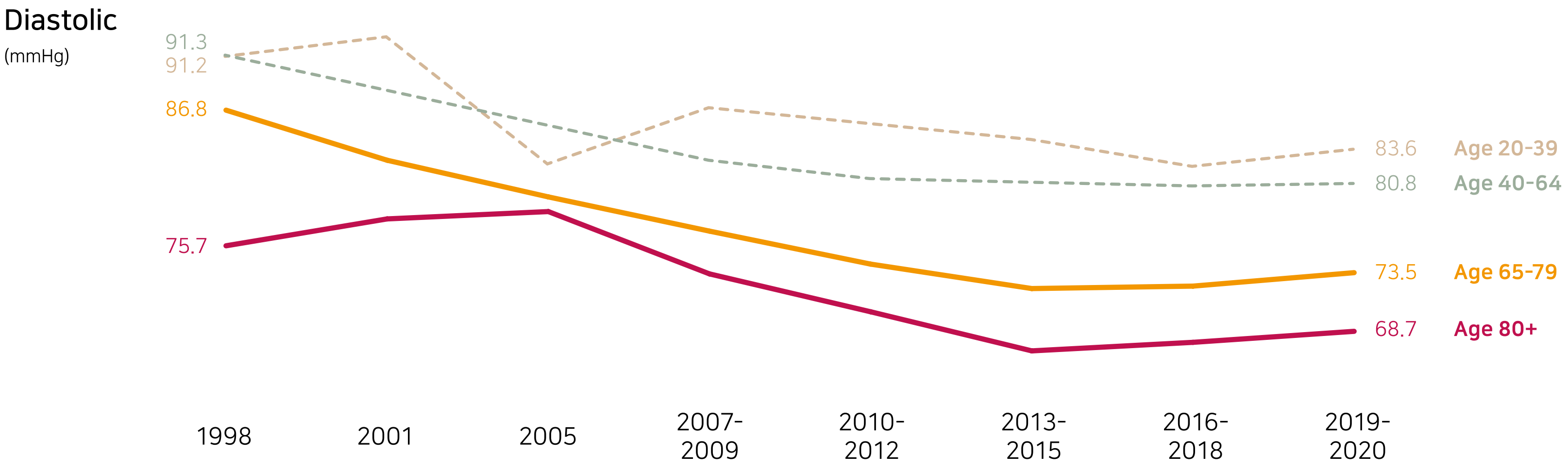

Data Source: Korea National Health and Nutrition Examination Survey 1998-2020

KOREA HYPERTENSION FACT SHEET 2022

This fact sheet will be published in Clinical Hypertension, the official journal of the Korean Society of Hypertension
The Korean Society of Hypertension (KSH) - Hypertension Epidemiology Research Working Group.
Korea Hypertension Fact Sheet 2022. Clinical Hypertension 2022 (in press).

www.koreanhypertension.org
