# Supplementary material for: Korea hypertension fact sheet 2022: analysis of nationwide population-based data with a special focus on hypertension in the elderly
Source: Clin Hypertens. 2023 Aug 15;29:22. doi: 10.1186/s40885-023-00243-8 (PMC10426053; doi:10.1186/s40885-023-00243-8)
Supplement: Supplementary file 2 — Supplementary Material 2 [file 40885_2023_243_MOESM2_ESM.docx]

Response Statement:

Thank you for the editor and reviewers’ valuable comments on our manuscript. We appreciate your thoughtful comments and suggestions, which have helped us improve our research's clarity, consistency, and completeness. We carefully considered all the comments to revise the manuscript.

Reviewer #1: In the present study entitled " Korea Hypertension Fact Sheet 2022: analysis of nationwide population-based data with a special focus on hypertension in the elderly", authors aimed to provide an overview of the magnitude and management status of hypertension and their recent trends in Korea. For this purpose, authors analyzed two nationally representative datasets, the Korea National Health and Nutrition Examination Survey (KNHANES) from 1998 to 2020 and the National Health Insurance (NHI) Big Data from 2002 to 2020.

This is a valuable and informative paper to understand the current status of hypertension and hypertension treatment in Korea. This paper is a well-written, well-illustrated and clear. I have only few questions and recommendation.

1. Authors used the operational definition of hypertension using I10 code for the analysis of NHI Big Data to maintain the comparability of Korea Hypertension Fact Sheet. However, many older hypertensive patients had hypertensive complications or target organ damages, thus I10 code (essential hypertension) is not sufficient to reflect the burden of hypertension especially for the older patients. This limitation should be presented in the discussion section.

[RESPONSE] Thank you for acknowledging the limitation of using the I10 code as an operational definition for hypertension in our study. We appreciate your suggestion to include this limitation in the discussion section of our manuscript. In the revision, we described this limitation in detail, highlighting that the I10 code may not capture the full burden of hypertension, particularly among older patients with hypertensive complications or target organ damages. Thank you for bringing this to our attention and helping us improve the clarity and accuracy of our study.

We added the following content to the revised manuscript:

*“…Second, using the I10 code as an operational definition of hypertension was intended to maintain comparability across series-issued Hypertension Fact sheets. But we acknowledge that this approach has limitations for identifying people with hypertension, particularly for older patients with hypertensive complications or target organ damage…”*

2. The prevalence of combination therapy decreased among the older patients aged 65-79 during the previous 10 years (page 35). Please discuss further regarding this finding.

[RESPONSE] Thank you for your insightful comment and for raising a question regarding the decrease in the prevalence of combination therapy among older patients aged 65-79 over the past ten years. The data shows that the prevalence of combination therapy in older adults treated for hypertension peaked at about 62% between 2022 and 2012 before falling below 60%. One possible explanation for this trend could be the increasing number of older patients with hypertension who are consistently adherent to their antihypertensive medication regimen, which could lead to sufficient blood pressure control with even a single antihypertensive drug. Another possible explanation could be the increasing emphasis on tailored treatment plans for older patients with multiple comorbidities. This may have led to more cautious prescribing of combination therapy due to concerns about potential drug interactions and adverse effects. Recent hypertension treatment guidelines for older patients, particularly the very elderly, have recommended different blood pressure targets, which may have contributed to changes in medication use patterns. However, further analysis is needed to fully understand the factors contributing to decreased combination therapy use among older patients. Future studies should consider factors such as blood pressure level before starting medication, the degree of blood pressure reduction after treatment, the dosage and duration of medication use, and potential side effects and adverse events to identify the specific factors driving changes in medication use patterns among older patients with hypertension.

We added the following content to the revised manuscript:

*“…Although the population average blood pressure and the prevalence of hypertension have remained relatively stable over the last decade, the absolute number of people with hypertension has increased steadily and now exceeds 12 million due to population aging. Of particular concern is the rapidly increasing number of elderly individuals with hypertension, with the number of very elderly women with hypertension increasing even faster. This trend may lead to a significant increase in the burden of hypertension and its complications. Fortunately, there have been improvements in the awareness, treatment, and treatment adherence rates among this age group. However, it is worth noting that the proportion of combination therapy use has recently decreased among older adults taking antihypertensive medications. One possible explanation for this trend is that increasing adherence to antihypertensive medication regimens may have led to sufficient blood pressure control with a single antihypertensive drug. Another possible explanation is that the target blood pressure has become more diverse among older patients. However, to fully understand the factors contributing to the decrease in combination therapy use among older patients, further studies with more detailed clinical information will be needed…”*

3. In page 7, 157-159: "However, it is also worth mentioning the recent decrease in awareness, treatment, and control rates among male hypertension patients aged 80 or older (Supplement, page 32-34)." The figures show the overall trends among all older patients. Please add male and female data if there is any significant gender difference in the awareness, treatment, and control rates.

[RESPONSE] Thank you for bringing to our attention that we have cited the wrong page numbers for the supplement where the data on sex and age-specific awareness, treatment, and control rates are presented. We apologize for any confusion this may have caused. The sex and age-specific data on awareness, treatment, and control rates can be found in the supplement on pages 16-20, not 32-34 as we originally cited.

Reviewer #2: It is exceptionally important paper, worth rapid publishing.

[RESPONSE] Thank you for your positive feedback and for recognizing the importance of our research. We appreciate your recommendation for rapid publishing and will work to ensure that the manuscript is ready for publication as soon as possible.
